# Screening of Q-markers for the wine-steamed *Schisandra chinensis* decoction pieces in improving allergic asthma

**DOI:** 10.1186/s13020-023-00712-0

**Published:** 2023-01-30

**Authors:** Zhongyuan Qu, Yifan Bing, Tianlei Zhang, Yan Zheng, Shuang Wu, Chenfeng Ji, Wenlan Li, Xiang Zou

**Affiliations:** 1 School of Pharmacy, Harbin University of Commerce, Harbin, 150076 China; 2 Engineering Research Center on Natural Antineoplastic Drugs, Ministry of Education, Harbin University of Commerce, Harbin, 150076 China; 3 School of Life Sciences, University of Sussex, Brighton, BN19RH UK

**Keywords:** Wine-steamed *Schisandra chinensis*, Decoction pieces, Quality markers, Allergic asthma, UPLC-Q-TOF-MS/MS

## Abstract

**Background:**

Traditional Chinese medicine (TCM) posits that Chinese medicinal materials can only be clinically used after being processed and prepared into decoction pieces. Schisandra Chinensis Fructus (derived from the dried and mature fruits of *Schisandra chinensis* (Turcz.) Baill.) has been used as a traditional antiasthmatic, kidney strengthening, and hepatoprotective agent for 2000 years. The results of previous research show that decoction pieces of wine-steamed Schisandra chinensis (WSC) are more effective than raw decoction pieces of Schisandra chinensis (RSC) for treating cough and asthma. Steaming with wine was demonstrated to promote the dissolution of ingredients. However, the relationship between the changes in the components of the decoction pieces of WSC and the therapeutic effect remains unclear.

**Methods:**

The efficacies of decoctions of RSC and WSC were compared using allergic asthma rats. The potential bioactive components in the serum of the WSC treatment group and the changes in the chemical composition of the RSC decoction pieces before and after wine steaming were determined by ultra-performance liquid chromatography quadrupole time-of-flight mass spectrometry (UPLC-Q-TOF-MS/MS) and ultra-high-performance liquid chromatography tandem mass spectrometry (UPLC H-CLASS XEVO TQD) to speculate quality markers (Q-markers) related to the efficacy of WSC, which were subsequently verified based on a zebrafish inflammation model.

**Results:**

Steaming RSC decoction pieces with wine was found to promote improvement of allergic asthma. Reverse tracing of 22 components detected in the serum of the high dose group of WSC (WSC-H) resulted in 12 ingredients being finally designated as potential effective components. Among these ingredients, 5 components, Schisandrin, Schisandrol B, Schisandrin A, Schisandrin B, and Gomisin D, had higher dissolution rates than RSC after steaming with wine. Validation by an inflammatory zebrafish model showed that these 5 ingredients had a dose-dependent effect and were therefore Q-markers for WSC in the treatment of allergic asthma.

**Conclusion:**

In this study, changes in the components of decoction pieces of RSC and WSC and Q-markers related to WSC efficacy were identified, providing valuable information for expanding the application of WSC and establishing a specific quality standard for WSC.

**Supplementary Information:**

The online version contains supplementary material available at 10.1186/s13020-023-00712-0.

## Introduction

Schisandra Chinensis Fructus (the dried and mature fruits of *Schisandra chinensis* (Turcz.) Baill.) was first recorded in *Shennong's Herbal Classic of Materia Medica* and is widely applied in the treatment of asthma, liver disease, and insomnia [1–3]. In addition to 12 ancient prescriptions for the treatment of cold pathogenic and miscellaneous diseases containing RSC recorded in the *Compendium of Materia Medica* (a classic treatise published approximately 2000 years ago that is regarded as having seminal pharmacological value), there is increasing evidence that RSC has curative effects on asthma [4, 5]. Drugs commonly used to treat asthma clinically, such as inhaled corticosteroids and long-acting bronchodilators, often have prominent side effects [6]. However, RSC possesses the advantages of few toxic side effects, while acting synergistically on multiple targets, making research on RSC very valuable. Previous studies on RSC have led to the discovery of several kinds of constituents, including dibenzocyclooctadiene lignans [7], organic acids [8], polysaccharides [9], and volatile oils [10]. The most biologically effective of these components are lignans, such as Deoxyschizandrin, Schisantherin A, Schisandrin B, and Schisandrin, with antitussive, expectorant, and anti-inflammatory effects [11].

TCM decoction pieces refer to the finished product of processing original Chinese medicinal materials according to clinical preparation requirements of TCM under the guidance of TCM theory. Processing is one of the characteristics of TCM and has the function of reducing toxicity, strengthening bioactivity, or modifying the nature of medicinal materials. Some of the most commonly used processing methods include stir-frying with vinegar or wine and steaming with water or rice wine [12, 13]. Many chemical reactions, such as hydrolysis, oxidation, and decomposition, are considered to occur between medicinal materials and auxiliary materials during processing [14], which causes the difference in efficacy between original medicinal materials and processed pieces. Commonly used processed products of RSC include wine-, honey-, and vinegar-steamed products. According to TCM theory, yellow rice wine has strong antioxidant activity that can promote the dissolution and absorption of ingredients and improve their curative effect [15]. The warming and tonifying effect of RSC is enhanced by steaming with wine, and RSC has been included in the classic clinical tonics of "Wuzi Bushen Pill" and "Maiwei Dihuang Pill", in line with the guidelines of “use as a tonic after being processed”. In addition, an allergic asthma attack is related to deficiencies of Qi and Yang, and the long-term course and repeated attacks of this disease damage internal organs. WSC has a strong tonic effect that is speculated to alleviate allergic asthma. In previous studies by our research group, WSC was demonstrated to treat allergic asthma more effectively, as well as producing a stronger antitussive effect than honey-steamed RSC. However, there are few reports on treating allergic asthma with WSC.

**Fig. 1:**
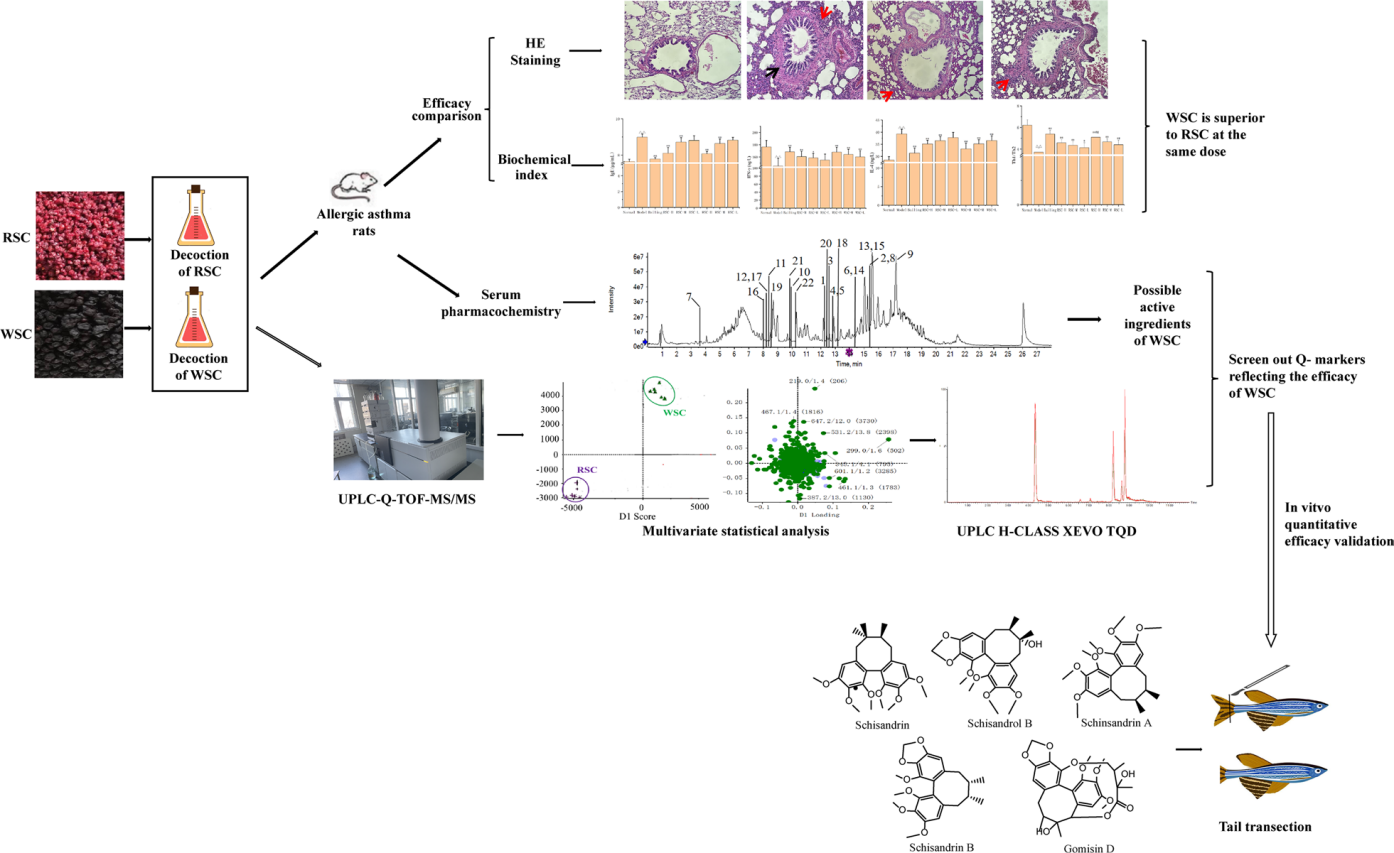
Graphical abstract for the entire study

Decoction is one of the most common forms of Chinese medicine administered clinically administration that produces remarkable curative effects and has been used for thousands of years. The clinical efficacy of a decoction results from the main components of the decoction. Many scholars have conducted systematic analyses of the chemical components in the ethanol extract of RSC [16, 17]. However, it is still unclear which components can be extracted from the decoction and which components can be absorbed by blood during treatment, making it difficult to establish a specific quality evaluation standard for decoction pieces. Therefore, to investigate the efficacy of WSC in treating allergic asthma and establish a specific quality evaluation standard for WSC decoction pieces, we screened the active components of WSC decoction pieces using an allergic asthma model, determined the Q-markers related to WSC efficacy by identifying differences in the compositions of RSC and WSC decoction pieces, and verified the screening results for the Q-markers using a zebrafish model. A graphical abstract is shown in Fig. 1.

## Materials and methods

### Materials and reagents

Eight standard compounds in Schisandrae Chinensis Fructus with a purity > 98% were obtained from the Chengdu Institute of Biology, Chinese Academy of Sciences (Chengdu, China), including Schisandrin A, Schisandrin B, Schisanhenol, Gomisin D, Schisandrol B, Schisandrin, Schisantherin B and Schisantherin A. OVA was purchased from the Beijing Boao Biological Co., Ltd. (Beijing, China). Aluminum hydroxide and Bailing capsules were purchased from the Hangzhou Zhongmei Huadong Pharmaceutical Co., Ltd. (Hangzhou, China). HPLC-grade acetonitrile and methanol were provided by Merck (Darmstadt, Germany). Formic acid (HPLC-grade) was collected from Thermo-Fisher (New York, US). The water used in the LC‒MS analysis was acquired from Watsons (Guangzhou, China). Dexamethasone (Dex) and tricaine were provided by Sigma‒Aldrich (Darmstadt, Germany). All the ELISA kits used in this study were produced by the Shanghai Guangrui Biotechnology Co., Ltd. (Shanghai, China).

### Animals

Ninety male SD rats weighing 180 ± 20 g were provided by the Changchun Yisi Experimental Animal Technology Co., Ltd. (Changchun, China; License No: 2018095). All the rats were randomly housed in temperature-controlled cages under 50% ± 10% humidity and a 12-h light–dark cycle. The rats were acclimated for one week before performing experiments that were approved by The Animal Ethics Committee of the School of Pharmacy, Harbin University of Commerce (HSDYXY2018025).

A total of 660 adult AB strain zebrafish were purchased from the National Zebrafish Resource Center and housed in a circular culture system in our laboratory at a water temperature of 28 ± 0.5 ℃ and a dark/photoperiod of 10 h/14 h, as stated in the Zebrafish Book [18].

### Preparation of RSC and WSC extracts

Schisandrae Chinensis Fructus was collected from Heilongjiang Province and confirmed by Professor Jin Zhexiong at the School of Pharmacy, Harbin University of Commerce. WSC was prepared as follows: RSCs were mixed with wine in a sealed container for 1 h (5:1. RSC: wine, M: V), steamed until the wine was absorbed completely and dried at 60 °C.

The dried RSC or WSC decoction pieces were soaked for 30 min. The first decoction was performed in a volume of water that was 10 times the RSC mass for 30 min, and the second decoction was performed in a volume of water that was 8 times the RSC mass for 20 min. The cooking liquid from the two decoctions was filtered through a silk cloth, combined and concentrated to 1 g/mL.

### Rat experiments

An allergic asthma model was induced according to a previous report [19]. Ninety SD rats were randomly selected and divided into 9 groups (n = 10): normal, model, positive drug (Bailing capsule, 0.92 g/kg/d), and high-, medium- and low-dose RSC and WSC groups. All rats, except those in the control group, were intraperitoneally injected with 1 mL/d of a suspension of sodium chloride solution (containing OVA (60 mg, 5%) and 100 mg (10%) of aluminum hydroxide gel) at 1 and 8 days to conduct sensitization tests. From Day 9 to Day 28, the rats that had been sensitized to OVA were challenged with 1% aerosolized OVA to construct the asthma model. The normal group was administered 0.9% normal saline daily. Drugs were administered to the treatment groups by gavage. The general behavior of the rats was observed 1 h after the last dose. Based on clinical research, RSC is often decocted at a dose of 6–15 g in the treatment of asthma and wheezing cough [20]. Therefore, a one-fold dose of 15 g was selected to set up 1/twofold, onefold, and twofold dose groups, corresponding to 0.77 g/kg, 1.54 g/kg, and 3.09 g/kg as the daily intake of rats, respectively. Therefore, the dosages of RSC and WSC in the high-, medium- and low-dose groups were 3.09 g/kg, 1.54 g/kg, and 0.77 g/kg, respectively.

### Enzyme-linked immunosorbent assay (ELISA)

Briefly, the rats were euthanized and 6 ~ 8-mL blood samples were collected to determine indicators and identify the components absorbed into the blood. Each specimen was centrifuged at 3000 rpm for 15 min at 4 °C, and the supernatant was collected for ELISA to quantify the contents of immunoglobulin E (IgE), interferon γ (IFN-γ), and interleukin 4 (IL-4).

### Histology staining

After the collection of blood from the rats, the rat lungs were removed for pathological examination. Briefly, the lungs were subjected to fixation in 10% formaldehyde solution, paraffin embedding, sectioning, and dewaxing, and then stained with hematoxylin–eosin (HE) for observation.

### Preparation of serum samples

To investigate the serum pharmacochemistry of the blank and WSC-H groups, the corresponding blood samples were treated with methanol and transferred to a UPLC-Q-TOF-MS/MS for analysis. Briefly, serum proteins were precipitated from the blood by methanol addition (using a serum: methanol ratio of 1:3) at 4 °C and centrifuged for 10 min at 4000 rpm. Next, the supernatant was evaporated under nitrogen at 35 ℃, dissolved in 200 μL of methanol, and passed through a filter (with a 0.22-μm pore size) for UPLC-Q-TOF–MS/MS analysis.

### Chromatographic and mass spectrometric conditions used for qualitative analysis

Chromatographic separation was carried out on a UPLC HSS T3 column (Waters, Milford, 1.8 μm × 100 mm × 2.1 mm, USA) using a 28-min gradient with a flow rate of 0.2 mL/min and an injection volume of 5 μL. The column temperature was 35 ℃. The mobile phase consisted of deionized water containing 0.1% formic acid (A) and acetonitrile (B). The gradient elution program was as follows: 0–3 min, 0–23%B; 3–7 min, 23–40%B; 7–8 min, 40–42%B; 8–11.5 min, 42–55%B; 11.5–13 min, 55–65%B; 13–14.5 min, 65–75%B; 14.5–19 min, 75–100%B; and 19–28 min, 100-23%B.

A mass analysis was performed on an Agilent G6545 Q-TOF mass spectrometer (Agilent, USA) in positive- and negative-ion modes within the mass range of *m/z* 80 ~ 1500. The optimized ESI conditions were as follows: collision energy, 35 eV (positive-ion mode) and -35 eV (negative-ion mode); curtain gas (N_2_), 35 psi; sheath gas (N_2_), 55 psi; auxiliary gas (N_2_), 5 psi; ion spray voltage, 5.5 kV (positive-ion mode) and 4.5 kV (negative-ion mode); and collision energy spread, 15 eV. The error limit of the retention time and mass was set to 0.01 min and 0.01 Da, respectively.

### Systematic analysis of metabolites in serum samples

A database containing English names, molecular formulas, molecular weights, and structural formulas of all the compounds and metabolic ingredients in RSC was established by reviewing the Chinese and English literature and searching PubChem, TCMSP, ChemSpider, and other databases. The original serum data for the WSC-H group obtained by the UPLC-Q-TOF-MS/MS analysis were imported into MakerView software, and standard conditions for data screening were used of an error limit for the retention time of 0.01 min and a mass error limit of 0.01 Da. The data for the screened blank and WSC-H groups were imported into SIMCA 14.1 software for multivariate statistical analysis. Then, the established database was imported into MakerView, and normal serum was used as the control to deduct the endogenous components to screen out the ones existed in the WSC-containing serum.

### UPLC-Q-TOF–MS/MS analysis of RSC and WSC extracts

Preparation of the mixed standard: Stock solutions of Schisandrin A, Schisandrin B, Schisanhenol, Gomisin D, Schisandrol B, Schisandrin, Schisantherin B, and Schisantherin A standards were prepared in acetonitrile at a concentration of 0.2 mg/mL. All samples were filtered through a 0.22-μm microporous membrane before use.

Preparation of decoction samples: The RSC and WSC samples obtained following the procedure described in the section “Preparation of RSC and WSC extracts” were concentrated and redissolved in 25 mL of highly pure acetonitrile to a concentration of 0.04 g/mL. UPLC-Q-TOF-MS/MS analysis was performed under the aforementioned chromatographic and mass spectrometric conditions.

Data analysis: The original RSC and WSC data obtained from the sample analysis were imported into MakerView and analyzed following the method used to analyze the serum sample data.

### Multivariate statistical analysis to determine differences between components of RSC and WSC decoction pieces

The aim of principal component analysis (PCA) is to extract a few principal components from multiple indicators through dimensionality reduction, while retaining as much information as possible. Specifically, six samples of the same batches of RSC and WSC were prepared, and the obtained sample data were imported into MakerView for analysis to produce PCA score plots and loading plots. The self-built chemical composition database of RSC and *VIP* > 1 as the reference index were used in conjunction with a T test to screen for compounds with significant differences (*P* < 0.05), with the parameters of the mass error, secondary fragment and isotope similarity ratio as the identification basis.

### Quantitative verification based on UPLC H-CLASS XEVO TQD

#### Preparation of decoction samples

Samples were prepared following the procedure described in the section “Preparation of RSC and WSC extracts”. The filtered prepared solution was concentrated to dryness and diluted to 10 μg/mL by adding the requisite volume of methanol, followed by filtration through a 0.45-μm microporous membrane.

#### Preparation of methanol extraction samples

RSC and WSC samples were accurately weighed (0.32 g) and placed in 25-ml measuring bottles, which were then filled with methanol. The samples were extracted by ultrasonication (250 W, 20 kHz) for 20 min, the requisite quantity of methanol was added to the samples, and the resulting solutions were filtered. Finally, an appropriate quantity of filtrate was diluted to 10 μg/mL with methanol and filtered through a 0.45-μm microporous membrane.

#### Standard preparation

Appropriate quantities of Schisandrin, Schisandriol B, Schisandrin A, Schisandrin B, Schisanhenol, and Gomisin D were accurately weighed, placed in 10-mL bottles and dissolved in methanol to obtain mixed reference stock solutions with mass concentrations of 0.64 μg/mL, 0.08 μg/mL, 0.2 μg/mL, 0.33 μg/mL, 0.025 μg/mL, and 0.021 μg/mL, respectively. The samples were diluted 1, 2, 4, 8, 16, and 32 times and reserved until use.

#### Chromatographic and mass spectrometric conditions

Chromatographic analysis was performed using a UPLC HSS T3 column (Waters, Milford, 1.8 μm × 100 mm × 2.1 mm, USA) at 35 ℃. The mobile phase was 0.1% formic acid (A) and acetonitrile (B), and the elution gradient program was as follows: 0–2 min, 40–52% B; 2–6 min, 52–75% B; 6–8 min, 75–90% B; 8–10 min, 90–40% B; and 10–12 min, 40–40% B. The volumetric flow rate was 0.2 mL/min, and the sample injection volume was 2 μL. Mass spectrometry detection was carried out by using an ESI in positive-ion mode and for MRM. The flow rates of the curtain and sheath gases were 35 psi and 55 psi, respectively. The spray voltage was 5.5 kV. The specific parameters of the mass spectra of the 6 components are shown in Additional file 2: Table S1.

#### Method validation

The method was validated in terms of the linearity, precision, stability, repeatability, and sample recovery. The linearity was evaluated by determining the contents of the mixed reference solutions at 6 different concentrations. The precision was determined by injecting the same test solution 6 times consecutively on the same day. The stability was evaluated by analyzing the same test solution at 0, 2, 4, 8, 12, and 24 h. The repeatability was assessed by determining the contents of 6 test solutions prepared from WSC powder. The recovery rate was calculated by analyzing a solution with a known content to which the reference solution was added in a 1:1 ratio.

#### Determination of the water dissolution rate

The samples were analyzed by chromatography-mass spectrometry under the aforementioned conditions, and the contents of the 6 components in the samples were calculated according to the corresponding linear relationship. The dissolution rate was calculated using the Press formula (water dissolution rate = content of decoction/methanol extraction content × 100%).

### Validation of monomeric components based on a zebrafish inflammation model

Determination of the maximum tolerance concentration (MTC): 360 healthy adult zebrafish with similar body sizes were randomly selected and divided into a normal group and groups treated with RSC, WSC, Schisandrin, Schisandrol B, Schisandrin A, Schisandrin B, and Gomisin D. The RSC and WSC groups were treated at 390 μg/mL, 780 μg/mL, 1560 μg/mL, 3120 μg/mL, and 6240 μg/mL, respectively. The Schisandrin, Schisandrol B, Schisandrin A, Schisandrin B, and Gomisin D treatment groups were each divided into 5 groups treated at 10 20, 40 , 8, and 160 μM, with 10 fish in each group. The fish were cultured for 24 h, and the number of deaths in each group was counted to determine the MTC. The MTC and 1/2 MTC were used as the high and low doses for the RSC and WSC treatment groups. The MTC, 1/2 MTC, and 1/4 MTC were set as the high, medium, and low doses for each monomer administration group.

Tail transection and screening of administration time points: A total of 50 adult zebrafish were divided into 5 groups of 10 fish each, including one normal group and four model groups (with tail transections for 1, 2, 4, and 6 h). The model groups were anesthetized in water containing 0.1% tricaine, and the tail of each fish was cut off with a sterile scalpel on a triangular plate covered with 2% agarose (to prevent injury to the spinal cord) [21]. The amputated zebrafish were placed in water at 28.5 ℃, and culturing was continued for the requisite time. At the end of the experiment, 10 zebrafish in each group were anesthetized in an ice-water bath, and the fish tissue was cut with sterile surgical scissors and added to precooled 0.9% normal saline in a 1:9 ratio (m/m) in a homogenizer for grinding. The prepared homogenate was centrifuged at 2500 r/min for 10 min to obtain supernatants for determining the expression levels of tumor necrosis factor-α (TNF-α), interleukin 6 (IL-6), and interleukin 10 (IL-10). The time point at which the highest expression of inflammatory factors was obtained was used in subsequent studies.

Validation of the efficacy of RSC and WSC: A total of 70 adult zebrafish were divided into 7 groups of 10 fish each: normal, model, positive drug (10 μM Dex [22]), and high- and low-dose RSC and WSC groups. The zebrafish inflammation model was established through tail amputation for all groups except the normal group. The amputated zebrafish were treated with drugs for 2 h, and the levels of TNF-α, IL-6, and IL-10 in the fish were measured.

Efficacy validation: A total of 180 adult zebrafish were divided into 18 groups with 10 for each: the normal group, model group, positive drug group (10 μM Dex), high, medium, and low dose groups of each component. The same procedures were used to establish the model and measure indicators as those described in the section "Validation of the efficacy of RSC and WSC".

### Data analysis

Statistical analyses were conducted using SPSS software v21.0 (SPSS Corporation, Chicago, Illinois, US) and presented as the mean ± SD. The Shapiro‒Wilk test was used to verify whether the data followed a normal distribution. Data conforming to a normal distribution were further analyzed using one-way ANOVA to assess the difference in variability among groups.

## Results

### WSC had a more potent effect on allergic asthma than RSC at the same dose

After the last dose was administered to the rats, the general behavior of all groups was monitored. OVA atomization led to allergic asthma, which manifested as classical shortness of breath, panting, wheezing, and sneezing, indicating the successful establishment of allergic asthma. Treatment administration had an ameliorative effect on the symptoms mentioned above for the RSC and WSC groups, similar to that for the Bailing capsule group, which was mainly reflected in brighter fur, gradual weight gain, and an improved mental state. The rats in the RSC-H and WSC-H groups were in a better state than the other groups.

**Fig. 2:**
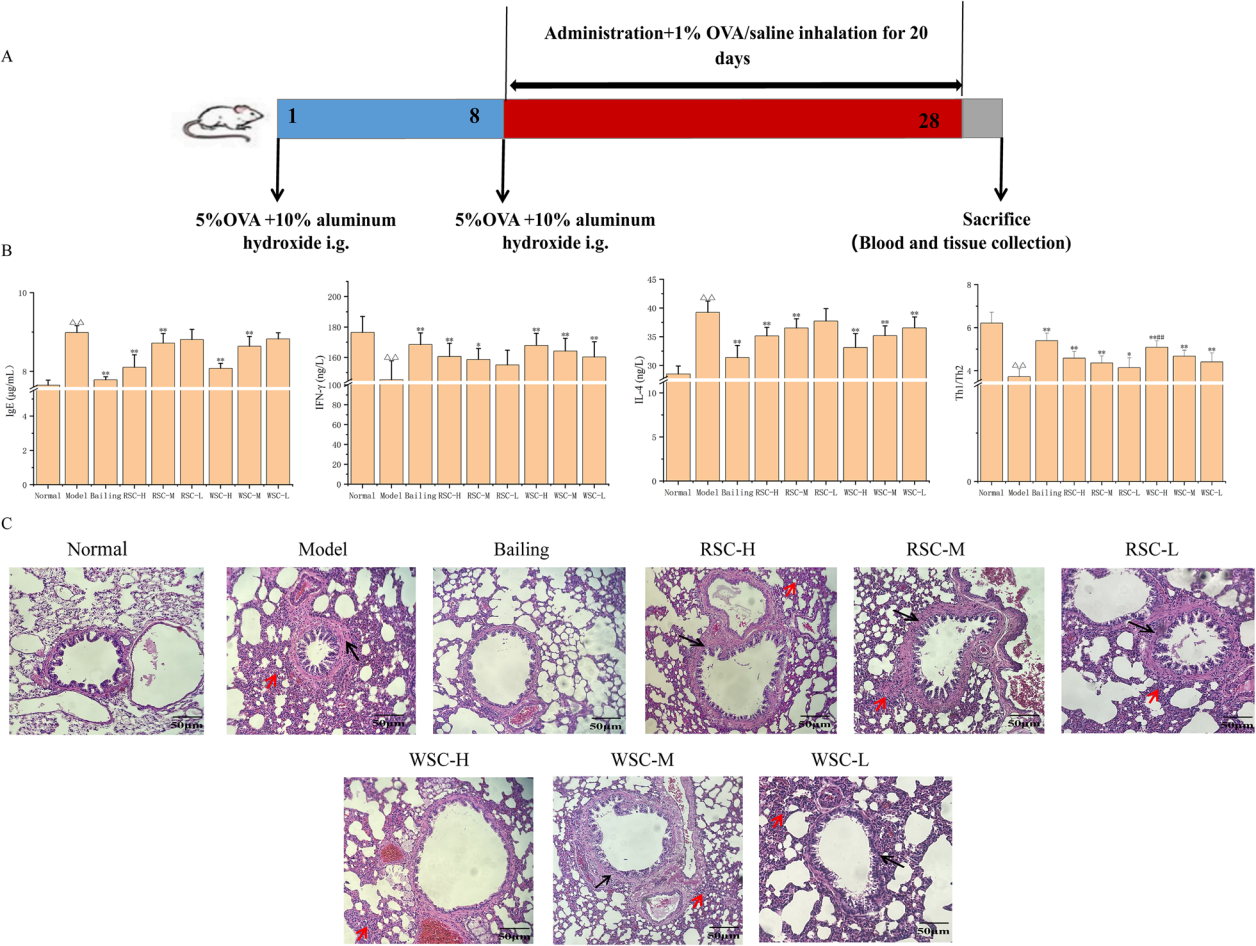
Amelioration effects of RSC and WSC on allergic asthmatic rats. **A** Establishment of the allergic asthma model. **B** Effects of the RSC and WSC decoctions on biochemical indices related to allergic asthma of treatment groups. The data are expressed as the mean ± SD (n = 10). Normal, normal group; Model, model group; Bailing, positive drug group; RSC-H, group treated with a high RSC dose; RSC-M, group treated with a medium RSC dose; RSC-L: group treated with a low RSC dose; WSC-H, group treated with a high WSC dose; WSC-M, WSC group treated with a medium WSC dose; WSC-L, group treated with a low RSC dose. ^△^*p* < 0.05 and ^△△^*p* < 0.01 vs. normal group, *p < 0.05 and **p < 0.01 vs. model group, ^#^*p* < 0.05 and ^##^*p* < 0.01 vs. WSC-H group. **C** Effects of the RSC and WSC decoctions on the lung histomorphology (red arrows indicate inflammatory infiltration, and black arrows indicate bronchial wall thickening; H&E × 40)

Compared to the results for the normal group, in the model group, the serum IFN-γ levels were distinctly lower (*P* < 0.01, Fig. 2B) and the expression of IL-4 and IgE was higher (*P* < 0.01, Fig. 2B), indicating that an imbalance in the Th1/Th2 ratio in allergic asthma rats. Both RSC and WSC administration caused an elevation in IFN-γ and a decrease in IL-4 and IgE levels in a dose-dependent manner compared with those of the model group. The highest treatment efficacy was observed for the RSC-H, WSC-H and WSC-M groups (*P* < 0.05 or *P* < 0.01). Note that WSC was more effective than RSC in increasing asthma-related decreased IFN-γ expression and reducing the asthma-related increased IL-4 content at the same dose, but these differences were not significant. However, an analysis of the differences in the Th1/Th2 ratio across groups showed that the Th1/Th2 ratio was significantly higher in the WSC-H group than in the RSC-H group (*P* < 0.01, Fig. 2B). The Th1/Th2 ratio was also higher in the WSC treatment group than in the RSC at other doses (no significant difference). RSC and WSC had almost the same regulatory effect on the IgE index.

In the normal group, the lung tissue was intact with no inflammatory cell infiltration around the airway, and the mucosa was free of proliferation and deformation. However, the rats in the model group exhibited inflammatory cellular infiltration, alveolar wall telangiectasia, and bronchial wall thickening (Fig. 2C). Treatment alleviated the detrimental effect of allergic asthma on the lung, as demonstrated by the remission of the infiltration of pulmonary inflammatory cells in the lung. Treatment had a more prominent ameliorative effect on the lung tissues in the WSC group than in the RSC group at the same dose.

### Optimization of the sample processing method

To determine the optimal serum pretreatment method, the results of precipitation using methanol and acetonitrile were compared. In positive-ion mode, 22 compounds were detected using methanol treatment compared to only 13 compounds using acetonitrile treatment (Additional file 1: Fig. S1). Although no corresponding compounds were detected in negative-ion mode, the total ion chromatogram of the methanol-treated serum contained more information than that of the acetonitrile-treated serum (Additional file 1: Fig. S2). Therefore, the methanol precipitation method was selected to treat the serum samples.

### Identification of potentially bioactive compounds of WSC

The efficacy comparison experiment carried out in this study proved that WSC had a more potent therapeutic effect on allergic asthma rats than RSC. To further explore the pharmacodynamic material basis of WSC, we adopted serum pharmacochemistry to screen the ingredients absorbed into the blood of the WSC-H group.

**Fig. 3:**
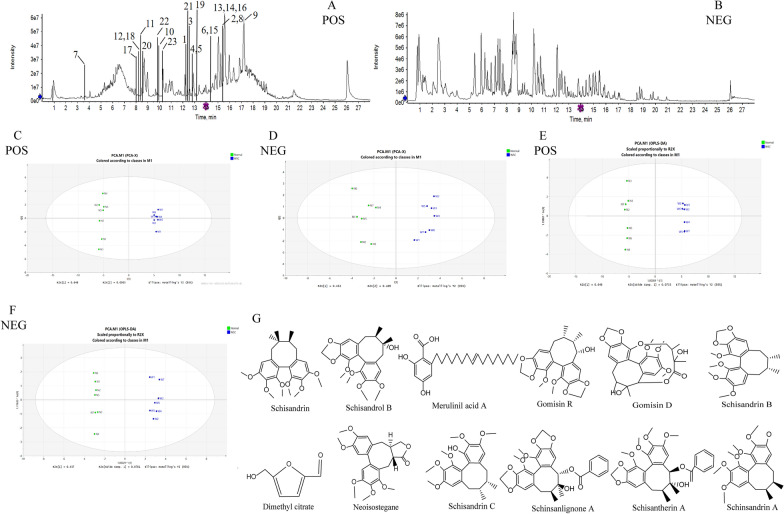
Identification of pharmacologically active components in the serum of the WSC-H group. **A** Total ion chromatogram of serum samples from the WSC-H group obtained in positive-ion mode. **B** Total ion chromatogram of serum samples from the WSC-H group obtained in negative-ion mode. The analysis conditions are detailed in the text, and the compounds labeled in the figure are listed in Table 1. **C** PCA diagram of the rat serum profile obtained in positive-ion mode. **D** PCA diagram of the rat serum profile obtained in negative-ion mode. **E** OPLS-DA diagram of the rat serum profile obtained in positive-ion mode. **F** OPLS-DA diagram of the rat serum profile obtained in negative-ion mode. **G** The structural formulas of the potentially bioactive compounds in serum

Specifically, the serum samples were analyzed by comparing the fragment pattern and retention time of the UPLC-Q-TOF–MS/MS data obtained in positive- and negative-ion modes (Fig. 3A, B. Figure 3C–F show the PCA and OPLS-DA loading diagrams of the serum metabolism spectrum of the normal and WSC-H groups obtained in positive- and negative-ion modes. The score chart shows the score points of the WSC drug-containing serum and the normal group were mainly distributed in two areas, indicating different information about the components was contained in the sample data.

**Fig. 4:**
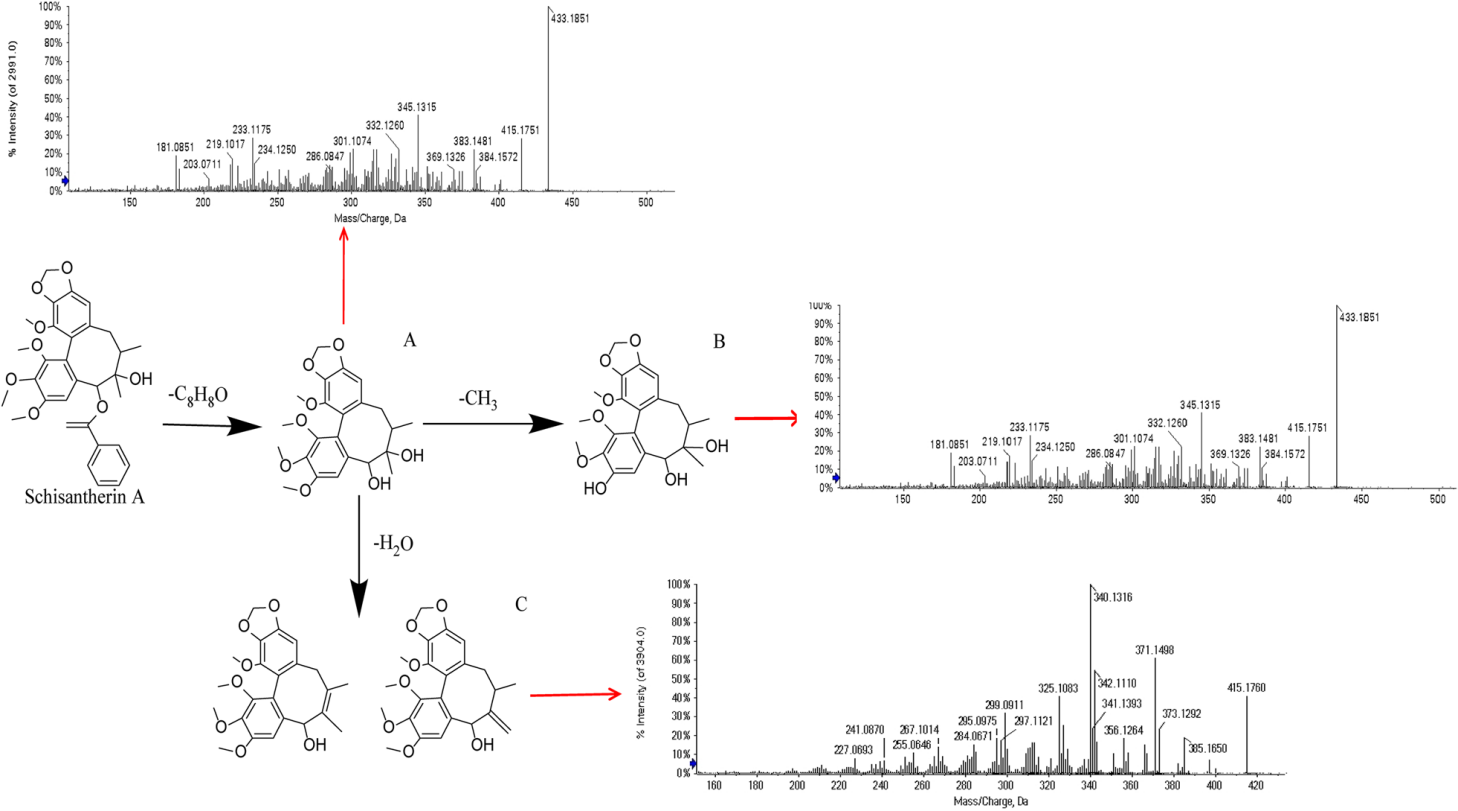
The proposed metabolic pathways of schisantherin A in serum. **A** The ion fragment of 6-debenzoyl-Schisantherin A. **B** The ion fragment of 3-demethylation-6-debenzoyl-Schisantherin A. **C** The ion fragment of 7,8-dehydration-6-debenzoyl-Schisantherin A or 7,17-dehydration-6-debenzoyl-Schisantherin A

A total of 22 compounds were detected in the serum of the WSC-H group, including 10 prototype components and 12 metabolites. Nine of these compounds, Schisandrin, Mercurial acid A, Gomisin R, Gomisin D, Schisandrin B, Dimethyl citrate, Neoiostegane, Schisandrin C, and Schisanlignone A, were detected in the serum completely in prototype form and 2 of these compounds, Schisanterin A and Schisandrin A, were transformed into 11 metabolites in serum. Furthermore, Schisandrin and one of its metabolites were also detected in serum. Therefore, although 22 compounds were detected in the serum, 12 components, including Schisandrin, Schisandrol B, Mercurial acid A, Gomisin R, Gomisin D, Schisandrin B, Dimethyl citrate, Neoiostegane, Schisandrin C, Schisanlignone A, Schisanterin A, and Schisandrin A, were finally designated as potential effective components of WSC based on the reverse traceability of prototype components and metabolites (Fig. 3G and Table 1). The metabolite of Schisantherin A is used as an example to illustrate the speculated cracking process based on reference [23], as shown in Fig. 4.

**Table 1:** Identification of prototype components and metabolites ingredients in serum of WSC

No	RT (min)	Adduction	*M/Z*	ppm	Formula	Fragments	Identification	Type
1	12.4	[M + H]^+^	433.2223	0.5	C_24_H_32_O_7_	433.1891 [M + H]^+^, 384.1905 [M + H-CH_4_O_2_]^+^, 369.1672 [M + H-CH_4_O_2_-CH_3_]^+^, 338.1488 [M + H-CH_4_O_2_-CH_3_-CH_3_O]^+^	Schisandrin	Prototype
2	15.4	[M + H]^+^	417.1909	0.5	C_23_H_28_O_7_	417.1815 [M + H]^+^, 399.1699 [M + H-H_2_O]^+^, 358.1321 [M + H-H_2_O-CH_3_-C_2_H_4_]^+^, 314.1084 [M + H-H_2_O-CH_3_-C_2_H_4_-CH_3_CO]^+^	Schisandrol B	Prototype
3	12.7	[M + H]^+^	391.2846	0.9	C_24_H_38_O_4_	391.3340 [M + H]^+^, 279.1769 [M + H-7CH_2_-CH_3_]^+^, 205. 1174 [M + H-7CH_2_-CH_3_-7CH_2_-2CH-COOH-2OH]^+^	Merulinic acid A	Prototype
4	12.9	[M + H]^+^	401.1598	1.1	C_22_H_24_O_7_	401.1602 [M + H]^+^, 352.1309 [M + H-OCH_3_]^+^, 337.1058 [M + H-OCH_3_-CH_3_]^+^	Gomisin R	Prototype
5	12.9	[M + H]^+^	531.2229	0.8	C_28_H_34_O_10_	531.2152 [M + H]^+^, 485.2161 [M + H-CH_2_O_2_]^+^, 401.1588 [M + H-CH_2_O_2_-C_5_H_8_O]^+^, 383.1488 [M + H-CH_2_O_2_-C_5_H_8_O-H_2_O]^+^	Gomisin D	Prototype
6	14.4	[M + H]^+^	401.1957	-0.2	C_23_H_28_O_6_	401.1957 [M + H]^+^, 370.1746 [M + H-OCH_3_]^+^, 331.1149 [M + H-C_5_H_10_]^+^, 316.0927 [M + H-CH_3_-C_5_H_10_]^+^, 300.0969 [M + H-CH_3_-O-C_5_H_10_]^+^	Schisandrin B	Prototype
7	3.62	[M + H]^+^	221.0652	− 1.5	C_8_H_12_O_7_	221.0655 [M + H]^+^, 157.0484 [M + H-OH-COOH-CH_3_]^+^,139.0023 [M + H-OH-COOH-CH_3_-H_2_O]^+^	Dimethyl citrate	Prototype
8	15.4	[M + H]^+^	415.1753	0.5	C_23_H_26_O_7_	415.1742 [M + H]^+^, 385.1644 [M + H-CH_2_O]^+^, 371.1481[M + H-CH_2_O-CH_3_]^+^	Neoisostegane	Prototype
9	17.3	[M + H]^+^	385.1646	0.1	C_22_H_24_O_6_	385.1650 [M + H]^+^, 355.1546 [M + H-OCH_3_]^+^, 285.0754 [M + H-OCH_3_-C_5_H_10_]^+^	Schisandrin C	Prototype
10	10.0	[M + H]^+^	431.2069	1.1	C_24_H_30_O_7_	431.2033 [M + H]^+^, 400.1865 [M + H-OCH_3_]^+^,372.1549 [M + H-OCH_3_-C_2_H_4_]^+^	Schinsanlignone A	Prototype
11	8.3	[M + H]^+^	433.1851	0.8	C_23_H_28_O_8_	433.1851 [M + H]^+^, 415.1751 [M + H-H_2_O]^+^, 384.1572 [M + H-H_2_O-OCH_3_]^+^, 369.1326 [M + H-H_2_O-OCH_3_-CH_3_]^+^, 345.1315 [M + H-H_2_O-C_5_H_10_]^+^, 301.1074 [M + H-H_2_O-CO_2_-C_5_H_10_]^+^ 286.0847 [M + H-H_2_O-CO_2_-C_5_H_10_-CH_3_]^+^	6-debenzoyl-Schisantherin A	Metabolites of Schisantherin A
12	8.1	[M + H]^+^	419.1709	0.5	C_22_H_26_O_8_	419.1709 [M + H]^+^, 401.1632 [M + H-H_2_O]^+^, 369.1361 [M + H-H_2_O-CH_3_OH]^+^, 331.1193 [M + H-H_2_O-C_5_H_10_]^+^, 316.0941 [M + H-H_2_O-C_5_H_10_-CH_3_]^+^	3-demethylation-6-debenzoyl-Schisantherin A	Metabolites of Schisantherin A
13	15.5	[M + H]^+^	415.1760	0.5	C_23_H_26_O_7_	415.1760 [M + H]^+^, 397.1634 [M + H-H_2_O]^+^, 385.1650 [M + H-OCH_2_]^+^, 371.1489 [M + H-OCH_2_-CH_3_]^+^, 342.1110 [M + H-OCH_2_-CH_3_-HCO]^+^, 340.1316 [M + H-OCH_2_-CH_2_-OCH_3_]^+^	7,8-dehydration-6-debenzoyl-Schisantherin A or 7,17-dehydration-6-debenzoyl-Schisantherin A	Metabolites of Schisantherin A
14	14.4	[M + H]^+^	401.1970	0.7	C_23_H_28_O_6_	401.1970 [M + H]^+^, 386.1740 [M + H-CH_3_]^+^, 370.1788 [M + H-OCH_3_]^+^, 369.1720 [M + H-CH_3_-OH]^+^, 355.1548 [M + H-2CH_3_-OH]^+^	2-demethoxy-Schisandrin A	Metabolites of Schisandrin A
15	15.5	[M + H]^+^	415.1760	0.5	C_23_H_26_O_7_	415.1760 [M + H]^+^, 397.1634 [M + H-H_2_O]^+^, 385.1650 [M + H-OCH_3_]^+^, 371.1498 [M + H-OCH_3_-CH_2_]^+^, 340.1316 [M + H-2OCH_3_-CH_2_]^+^, 325.1083 [M + H-2OCH_3_-CH_2-_COCH_3_]^+^	1-demethoxy-7,8-dicarbonylation-Schisandrin A	Metabolites of Schisandrin A
16	8.0	[M + H]^+^	419.1709	0.9	C_23_H_30_O_7_	419.1709 [M + H]^+^, 401.1632 [M + H-H_2_O]^+^, 387.1457 [M + H-H_2_O-CH_2_]^+^, 369.1361 [M + H-H_2_O-CH_2_-H_2_O]^+^, 327.1247 [M + H-H_2_O-CH_2_-4CH_3_]^+^,	3-demethyl-3,7-dihydroxy-Schisandrin A	Metabolites of Schisandrin A
17	8.1	[M + H]^+^	417.1917	0.7	C_23_H_30_O_8_	417.1917 [M + H]^+^, 399.1777 [M + H-H_2_O]^+^, 367.1546 [M + H-H_2_O-2OH]^+^, 331.1186 [M + H-H_2_O-2OH-2H_2_O]^+^, 313.1085 [M + H-H_2_O-2OH-3H_2_O]^+^	3-demethyl-3,7,8-trihydroxylated-Schisandrin A	Metabolites of Schisandrin A
18	13.2	[M + H]^+^	389.1957	0.6	C_22_H_28_O_6_	389.1957 [M + H]^+^, 374.1809 [M + H-CH_3_]^+^, 357.1713 [M + H-CH_3_-OH]^+^, 342.1463 [M + H-2CH_3_-OH]^+^, 329.1793 [M + H-CH_3_-OH-CO]^+^, 325.1441 [M + H-2CH_3_-OH-CO]^+^	3,12-dimethyldihydroxy-Schisandrin A	Metabolites of Shisandrin A
19	8.4	[M + H]^+^	433.1851	0.8	C_23_H_28_O_8_	433.1851 [M + H]^+^, 415.1751 [M + H-H_2_O]^+^, 384.1572 [M + H-H_2_O-OCH_3_]^+^, 369.1326 [M + H-H_2_O-OCH_3_-CH_3_]^+^	7-carboxylated -Schisandrin A	Metabolites of Schisandrin A
20	12.5	[M + H]^+^	415.2116	0.7	C_24_H_32_O_7_	415.2116 [M + H]^+^, 400.1888 [M + H-CH_3_]^+^, 384.1932 [M + H-OCH_3_]^+^, 369.1699 [M + H-OCH_3_-CH_3_]^+^, 338.1520 [M + H-2OCH_3_-CH_3_]^+^	7-hydroxylated -Schisandrin A	Metabolites of Schisandrin A
21	9.9	[M + H]^+^	449.3111	0.8	C_24_H_32_O_8_	449.3111 [M + H]^+^, 431.2076 [M + H-H_2_O]^+^, 382.1767 [M + H-H_2_O-2OH-CH_3_]^+^, 373.1656 [M + H-2OCH_3_-CH_3_]^+^, 358.1428 [M + H-2OCH_3_-2CH_3_]^+^	7,8-dihydroxy Schisandrin A	Metabolites of Schisandrin A
22	10.1	[M + H]^+^	432.3115	0.9	C_23_H_28_O_8_	432.3115 [M + H]^+^, 414.3028 [M + H-H_2_O]^+^, 357.2811 [M + H-CH_2_O_2_-CH_3_]^+^, 339.2707 [M + H-CH_2_O_2_-CH_3_-H_2_O]^+^, 321.2591 [M + H-CH_2_O_2_-CH_3_-2H_2_O]^+^	11-hydroxylated-Schisandrol B	Metabolites of Schisandrol B

The molecular ions at *m/z* 415.1751 [M + H-H_2_O]^+^, *m/z* 384.1572 [M + H-H_2_O-OCH_3_]^+^, *m/z* 369.1326 [M + H-H_2_O-OCH_3_-CH_3_]^+^, *m/z* 345.1315 [M + H-H_2_O-C_5_H_10_]^+^, *m/z* 301.1074 [M + H-H_2_O-CO_2_-C_5_H_10_]^+^ and *m/z* 286.0847 [M + H-H_2_O-CO_2_-C_5_H_10_-CH_3_]^+^ conformed to the fragmentation pattern of *m/z* 433.185. Based on these results, Compound 11 was identified as 6-debenzoyl-Schisantherin A [23].

The formula of Compound 12 was determined to be C_22_H_26_O_8_, which was only 14 Da lower in atomic mass than schisantherin A. Fragments of *m/z* 401.1632 [M + H-H_2_O]^+^, *m/z* 369.1361 [M + H-H_2_O-CH_3_OH]^+^, *m/z* 331.1193 [M + H-H_2_O-C_5_H_10_]^+^, and *m/z* 316.0941 [M + H-H_2_O-C_5_H_10_-CH_3_]^+^ were detected. Thus, Compound 12 was identified as 3-demethylation-6-debenzoyl-Schisantherin A [23].

Compound 13 was detected at 15.5 min, and its [M + H]^+^ ion at *m/z* 415.1760 was 18 Da lower in atomic mass than schisantherin A. The corresponding spectrum exhibited ions at *m/z* 397.1634 [M + H-H_2_O]^+^, *m/z* 385.1650 [M + H-OCH_2_]^+^, *m/z* 371.1489 [M + H-OCH_2_-CH_3_]^+^, *m/z* 342.1110 [M + H-OCH_2_-CH_3_-HCO]^+^, and *m/z* 340.1316 [M + H-OCH_2_-CH_2_-OCH_3_]^+^, which conformed to those of Schisantherin A. Hence, Compound 13 was identified as 7,8-dehydration-6-debenzoyl-Schisantherin A or 7,17-dehydration-6-debenzoyl-Schisantherin A [23]. The secondary mass spectra of these compounds are displayed in Fig. 4.

### Identification of common chemical compounds in RSC and WSC decoction pieces

**Fig. 5:**
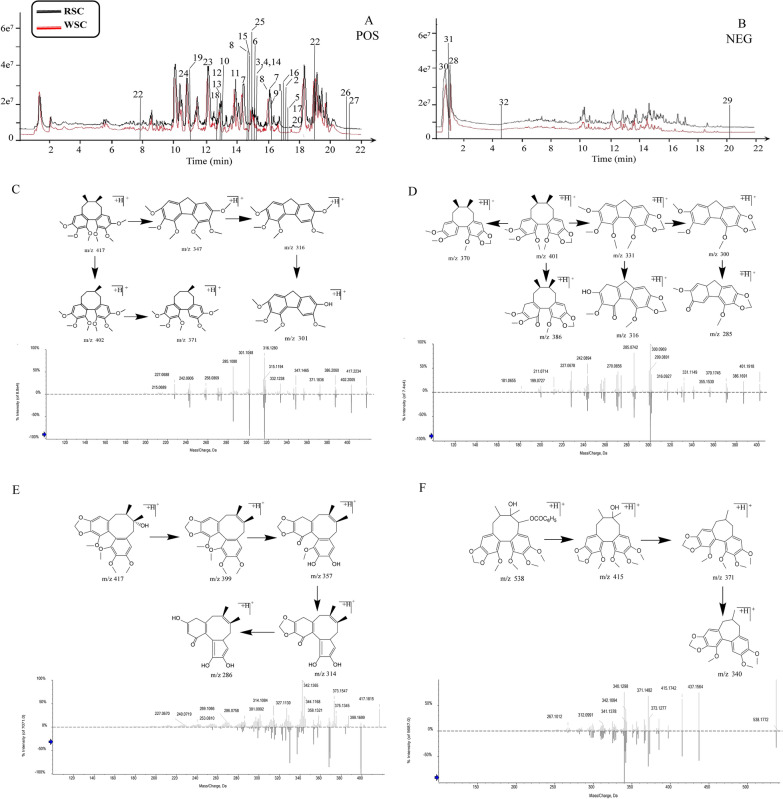
Comparison of chemical profiles of RSC and WSC. **A** Superposition of the total ion chromatograms of the RSC and WSC decoctions obtained in positive-ion mode, where the ingredients marked in the figure are listed in Table 2. **B** Superposition of the total ion chromatograms of the RSC and WSC decoctions obtained in negative-ion mode, where the ingredients marked in the figure are listed in Table 2. **C** The fragmentation pattern and secondary mass spectra of Schisandrin A. **D** The fragmentation pattern and secondary mass spectra of Schisandrin B. **E** The fragmentation pattern and secondary mass spectra of Schisandrol B. **F** The fragmentation pattern and secondary mass spectra of Schisantherin A

Analysis of the superposition diagram of the total ion flow patterns of RSC and WSC obtained in positive- and negative-ion modes (Fig. 5A, B) showed that no new components were produced by processing RSC. In addition, a total of 32 compounds, including 21 lignans and 11 organic acids, were identified as originating from the decoctions of RSC and WSC based on the retention time, fragmentation pattern, and precise mass measurements. Specific information for each compound is shown in Table 2.

**Table 2:** Identification of commom chemical compounds in the decoction of RSC and WSC decoction pieces

No.	RT (min)	Adduction	*M/Z*	ppm	Formula	Fragments	Identification	Type
1	16.9	[M + H]^+^	417.2237	− 8.1	C_24_H_32_O_6_	417.2234 [M + H]^+^, 402.2005 [M + H-CH_3_]^+^, 371.1836 [M + H-CH_3_-OCH_3_]^+^, 347.1465 [M + H-C_5_H_10_]^+^, 316.1280 [M + H-C_5_H_10_-OCH_3_]^+^, 301.1048 [M + H-CH_3_-OCH_3_-5CH_2_]^+^	Schisandrin A*	Lignan
2	17.4	[M + H]^+^	401.8561	− 5.7	C_23_H_28_O_6_	401.1957 [M + H]^+^, 370.1746 [M + H-OCH_3_]^+^, 331.1149 [M + H-C_5_H_10_]^+^, 316.0927 [M + H-CH_3_-C_5_H_10_]^+^, 300.0969 [M + H-CH_3_-O-C_5_H_10_]^+^	Schisandrin B*	Lignan
3	15.5	[M + H]^+^	417.1892	− 3.8	C_23_H_28_O_7_	417.1815 [M + H]^+^, 399.1699 [M + H-H_2_O]^+^, 358.1321 [M + H-H_2_O-CH_3_-C_2_H_4_]^+^, 314.1084 [M + H-H_2_O-CH_3_-C_2_H_4_-CH_3_CO]^+^	Schisandrol B*	Lignan
4	15.5	[M + H]^+^	537.2101	− 3.4	C_30_H_32_O_9_	538.1772 [M + H]^+^, 415.1742 [M + H-C_6_H_5_COOH]^+^, 371.1482 [M + H-C_6_H_5_COOH-CH_3_CHO]^+^, 340.1298 [M + H-C_6_H_5_COOH-CH_3_CHO-OCH_3_]^+^	Schisantherin A*	Lignan
5	17.6	[M + H]^+^	385.1646	0.1	C_22_H_24_O_6_	385.1650 [M + H]^+^, 355.1546 [M + H-OCH_3_]^+^, 285.0754 [M + H-OCH_3_-C_5_H_10_]^+^	Schisandrin C*	Lignan
6	15.3	[M + H]^+^	403.2098	− 4.3	C_23_H_30_O_6_	403.2080 [M + H]^+^, 388.1849 [M + H-CH_3_]^+^, 371.1827 [M + H-CH_3_-OH]^+^, 340.1645 [M + H-CH_3_-OH-OCH_3_]^+^, 325.1410 [M + H-2CH_3_-OH-OCH_3_]^+^	Schisanhenol*	Lignan
7	16.1	[M + H]^+^	515.2263	− 2.4	C_28_H_34_O_9_	515.2228 [M + H]^+^, 469.2183 [M + H–CO-H_2_O]^+^, 385.1615 [M + H–CO-H_2_O-C_5_H_8_O]^+^, 354.1441 [M + H–CO-H_2_O-C_5_H_8_O-OCH_3_]^+^	Schisantherin B	Lignan
8	14.8	[M + H]^+^	431.2033	− 5.6	C_24_H_30_O_7_	431.2033 [M + H]^+^, 400.1865 [M + H-OCH_3_]^+^, 372.1549 [M + H-OCH_3_-C_2_H_4_]^+^, 356.1598 [M + H-OCH_3_-C_2_H_4_-O]^+^	Schinsanlignone A	Lignan
9	16.17	[M + H]^+^	387.1790	− 3.2	C_22_H_26_O_6_	387.1786 [M + H]^+^, 372.1557 [M + H-CH_3_]^+^, 357.1676 [M + H-CH_3_-OH]^+^	Gomisin M_2_	Lignan
10	13.1	[M + H]^+^	389.1940	− 4.8	C_22_H_28_O_6_	389.1935 [M + H]^+^, 357.1678 [M + H-OCH_3_]^+^, 326.1500 [M + H-2OCH_3_]^+^	Gomisin J	Lignan
11	13.9	[M + H]^+^	391.2113	− 0.8	C_22_H_30_O_6_	391.2113 [M + H]^+^, 327.1602 [M + H-2OCH_3_]^+^, 257.0815 [M + H-2OCH_3_-C_7_H_6_O_2_]^+^	Pregomisin	Lignan
12	12.9	[M + H]^+^	401.1591	− 0.9	C_22_H_24_O_7_	401.1602 [M + H]^+^, 352.1309 [M + H-OCH_3_]^+^, 337.1058 [M + H-OCH_3_-CH_3_]^+^	Gomisin R	Lignan
13	12.9	[M + H]^+^	531.2206	− 3.5	C_28_H_34_O_10_	531.2152 [M + H]^+^, 485.2161 [M + H-CH_2_O_2_]^+^, 401.1588 [M + H-C_5_H_8_O]^+^, 383.1488 [M + H-C_5_H_8_O-H_2_O]^+^	Gomisin D	Lignan
14	15.5	[M + H]^+^	415.1742	− 4.4	C_23_H_26_O_7_	415.1742 [M + H]^+^, 385.1644 [M + H-CH_2_O]^+^, 371.1481 [M + H-CH_2_O-CH_3_]^+^	Neoisostegane	Lignan
15	14.5	[M + H]^+^	523.6521	− 4.0	C_30_H_34_O_8_	523.2287 [M + H]^+^, 493.1810 [M + H-2CH_3_]^+^, 409.1613 [M + H-2CH_3_-C_6_H_5_-2H_2_O]^+^	Benzoyl-gomisin-H	Lignan
16	17.0	[M + H]^+^	219.1733	− 4.7	C_15_H_22_O	219.1743 [M + H]^+^, 201.1632 [M + H-H_2_O]^+^, 175.1465 [M + H-H_2_O-2CH_3_]^+^	Chamigrenal	Organic acid
17	17.6	[M + H]^+^	521.2156	− 2.7	C_30_H_32_O_8_	521.2134 [M + H]^+^, 421.1615 [M + H-C_5_H_8_O_2_]^+^	Benzoylgomisin O	Lignan
18	12.4	[M + H]^+^	433.1891	− 2.9	C_24_H_32_O_7_	433.1891 [M + H]^+^, 384.1905 [M + H-CH_4_O_2_]^+^, 369.1672 [M + H-CH_4_O_2_-CH_3_]^+^, 338.1488 [M + H-CH_4_O_2_-CH_3_-CH_3_O]^+^	Schisandrin*	Lignan
19	11.2	[M + H]^+^	419.2052	− 2.9	C_23_H_30_O_7_	419.2062 [M + H]^+^, 369.1693 [M + H-CH_3_-2OH]^+^, 323.1276 [M + H-2CH_3_-2OH-CO]^+^	Gomisn S/T	Lignan
20	17.6	[M + H]^+^	537.2463	1.8	C_31_H_36_O_8_	537.2463 [M + H]^+^, 437.1931 [M + H-C_5_H_8_O_2_]^+^, 384.1933 [M + H-C_5_H_8_O_2_-H_2_O-OCH_3_]^+^	Benzoylgomisin Q	Lignan
21	2.4	[M + H]^+^	221.0655	2.3	C_8_H_12_O_7_	221.0655 [M + H]^+^, 157.0484 [M + H-OH-COOCH_3_]^+^, 139.0023 [M + H-OH-COOCH_3_-H_2_O]^+^	Dimethyl citrate	Organic acid
22	18.9	[M + H]^+^	471.3459	− 2.1	C_30_H_46_O_4_	471.3467 [M + H]^+^, 453.3241 [M + H-H_2_O]^+^, 435.3241 [M + H-2H_2_O]^+^	Nigranoic acid	Organic acid
23	12.0	[M + H]^+^	531.2594	1.0	C_29_H_38_O_9_	531.2596 [M + H]^+^, 471.2378 [M + H-2CH_2_O]^+^, 443.2426 [M + H–CO-2CH_2_O]^+^, 425.2342 [M + H–CO-2CH_2_O-H_2_O]^+^	Angeloylgomisin Q	Lignan
24	11.1	[M + H]^+^	499.2328	0.6	C_28_H_34_O_8_	499.2328 [M + H]^+^, 481.2217 [M + H-H_2_O]^+^, 463.2100 [M + H-2H_2_O]^+^, 421.2009 [M + H-2H_2_O-C_2_H_2_O]^+^	Angeloylisogomisin O	Lignan
25	15.2	[M + H]^+^	235.1722	3.2	C_15_H_22_O_2_	235.1722 [M + H]^+^, 217.1615 [M + H-H_2_O]^+^, 199.1513 [M + H-2H_2_O]^+^	β-chamigrenic acid	Organic acid
26	21.0	[M + H]^+^	455.3521	6.17	C_30_H_46_O_3_	455.3521 [M + H]^+^, 437.3411[M + H-H_2_O]^+^, 419.3304 [M + H-2H_2_O]^+^, 401.3363 [M + H-3H_2_O]^+^	Schisandronic acid	Organic acid
27	21.3	[M + H]^+^	391.3340	− 1.0	C_24_H_38_O_4_	391.3340 [M + H]^+^, 279.1769 [M + H-7CH_2_-CH_3_ + H]^+^	Merulinic acid A	Organic acid
28	1.2	[M-H]^+^	191.0187	− 3.2	C_6_H_8_O_7_	191.0187 [M-H]^+^, 173.0107 [M-H-H_2_O]^+^, 155.0016 [M-H-2H_2_O]^+^	Citric acid	Organic acid
29	20.1	[M-H]^+^	456.3606	4.7	C_30_H_48_O_3_	456.3606 [M-H]^+^, 409.3486 [M-H-COOH]^+^	Betulinic acidBetulinic acid	Organic acid
30	0.9	[M-H]^+^	133.0172	1.4	C_4_H_6_O_5_	133.0172 [M-H]^+^, 115.0065 [M-H-H_2_O]^+^, 114.6473 [M-H-H_2_O-H]^+^	Malic acid	Organic acid
31	1.1	[M-H]^+^	173.0093	0.8	C_7_H_10_O_5_	173.0093 [M-H]^+^, 129.0220 [M-H-2H_2_O-COOH]^+^ 111.0105 [M-H-3H_2_O-COOH]^+^	Shikimic acid	Organic acid
32	4.3	[M-H]^+^	291.0876	4.4	C_15_H_14_O_6_	291.0860 [M-H]^+^, 207.0649 [M-H-5OH]^+^, 189.0554 [M-H-5OH-H_2_O]^+^, 161.0601 [M-H-6OH-H_2_O]^+^	Catechin	Organic acid

*Ingredients to be compared with the standards

The lignans in RSC are mostly biphenyl cyclooctene lignans, which can be divided into three types according to the carbon substituents at Positions 2, 3, 6, 7, 12, 13, and 14. In the first type, there is no substituent at the carbons at Positions 6 and 7, such as in Schisandrin B. In the second type, there is a hydroxyl substituent at Position 7 and no substituent at Position 6, such as in Schisandrin B. In the third type, there is a hydroxyl substituent at Position 7 and an acyloxy substituent at Position 6, such as in Schisantherin A. The final data obtained by mass spectrometry showed that the lignans had a high response value using positive-ion mode. Most of the excimer ion peaks of the chemical components corresponded to [M + Na]^+^, [M + NH_4_]^+^, or [M + H]^+^.

The fragmentation pattern of the aforementioned three types of lignans is summarized. (1) Lignans without substituents at Positions 6 and 7 are inclined to lose methyl or methoxy groups, resulting in the fracture of the octatomic ring and the loss of C_5_H_10_ to form new ion fragments. (2) Lignans with a hydroxyl substituent at Position 7 and no substituent at Position 6 are generally dehydrated first and then mostly cleaved at the source and demethylated or lose a methoxy group to produce fragment ions. Alternatively, dehydration is followed by breakage of the biphenyl ring, loss of C_3_H_6_ and C_4_H_8_, and the production of new fragments. (3) For lignans with a hydroxyl substituent at Position 7 and an acyloxy substituent at Position 6, RCOOH can be easily removed from Position 6, followed by dehydration or removal of the CH_3_CHO group from Position 7. Schisandrin A, Schisandrin B, Schisandrol B, and Schisantherin A are used as examples to enumerate the analytical process in detail.

Compound 1 had a retention time of 16.9 min and a molecular formula of C_24_H_32_O_6_ and produced fragment ions at *m/*z 417.2234 [M + H]^+^, *m/z* 402.2005 [M + H-CH_3_]^+^, *m/z* 371.1836 [M + H-CH_3_-OCH_3_]^+^, *m/z* 347.1465 [M + H-C_5_H_10_]^+^, *m/z* 316.1280 [M + H-C_5_H_10_-OCH_3_]^+^, and *m/z* 301.1048 [M + H-CH_3_-OCH_3_-5CH_2_]^+^ in positive-ion mode. Based on the fragments of CH_3_ (15 Da), OCH_3_ (31 Da), C_5_H_10_ (70 Da), and OCH_3_ (31 Da), Compound 1 was preliminarily designated as Schisandrin A [24]. The fragmentation pathway was consistent with that reported in the literature. The possible cleavage fragmentation pathway of Schisandrin A is shown in Fig. 5C.

Compound 2 had a retention time of 17.4 min and a molecular formula of C_23_H_28_O_6_ and produced fragment ions at *m/z* 401.8561 [M + H]^+^, *m/z* 386.1691 [M + H-CH_3_]^+^, *m/z* 370.1745 [M + H-OCH_3_]^+^, *m/z* 331.1149 [M + H-C_5_H_10_]^+^, *m/z* 316.0927 [M + H-CH_3_-C_5_H_10_]^+^, *m/z* 300.0969 [M + H-CH_3_-O-C_5_H_10_]^+^, and *m/z* 285.1080 [M + H-CH_3_-C_5_H_10_-O-CH_3_]^+^ in positive-ion mode. Based on these fragments and the literature [26], the compound was identified as Schisandrin B. The specific possible fragmentation process is shown in Fig. 5D.

Compound 3 had a retention time of 15.4 min and an *m/z* of 417.1909 and was therefore speculated to be Schisandrol B (C_23_H_28_O_7_). There were four characteristic fragments in the map, including *m/z* 399.1699, *m/z* 357.1345, *m/z* 314.1084, and *m/z* 285.0758. The *m/z* 399.1699 [M + H-H_2_O]^+^ ion fragment lost CH_3_ and C_2_H_4_ to form the *m/z* 357.1345 [M + H-H_2_O-CH_3_-C_2_H_4_]^+^ ion fragment, then lost the CH_3_CO group to produce the *m/*z 314.1084 [M + H-H_2_O-CH_2_-C_2_H_4_-CH_3_-CO]^+^ ion fragment, and finally lost the methoxy group to produce the *m*/z 285.0758 [M + H-H_2_O-CH_2_-C_2_H_4_-CH_3_-CO-OCH_3_]^+^ ion fragment. Hence, Compound 3 was determined to be Schisandrol B [27]. The cleavage law and mass spectrum of Compound 3 are shown in Fig. 5E.

Compound 4 had a retention time of 15.4 min and a molecular formula of C_30_H_32_O_9_. Four fragments were detected in positive-ion mode at *m/z* 538.1772 [M + H]^+^, *m/z* 415.1742 [M + H-C_6_H_5_COOH]^+^, *m/z* 371.1482 [M + H-C_6_H_5_COOH-CH_3_CHO]^+^, and *m/z* 340.1298 [M + H-C_6_H_5_COOH-CH_3_CHO-OCH_3_]^+^. Compound 4 was determined to be Schisantherin A by comparing these results with those of the reference and literature [28]. The possible fragmentation patterns of Schisantherin A are presented in Fig. 5F.

### Analysis of the difference in the chemical constituents of RSC and WSC decoction pieces

Considering that wine steaming affects the chemical composition of medicinal materials and thereby, drug efficacy, the differences in the chemical components of decoctions of WSC and RSC were analyzed by UPLC-Q-TOF–MS/MS to identify possible Q-markers for the efficacy of WSC.

**Fig. 6:**
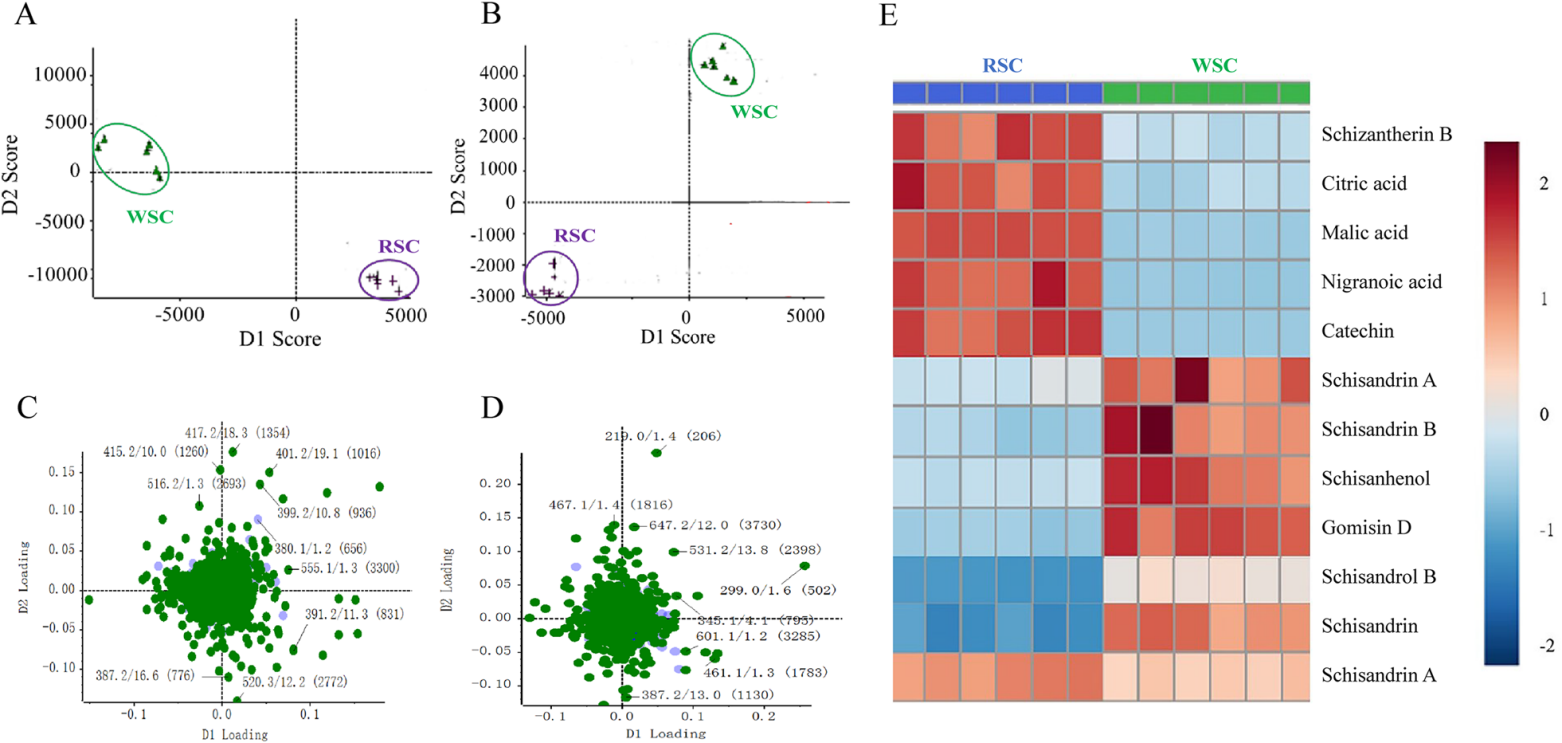
PCA of the RSC and WSC decoction pieces. **A** PCA score plot of the data for the RSC and WSC decoction pieces obtained in positive-ion mode. The data for the WSC decoction pieces were clearly separated from the data for the RSC decoction pieces. **B** PCA score plot of data for the RSC and WSC decoction pieces obtained in negative-ion mode. **C** S-plot for data of the RSC and WSC decoction pieces obtained in positive-ion mode. **D** S-plot for data of the RSC and WSC decoction pieces obtained in negative-ion mode. **E** Differences between the dissolution rates of the chemical components of RSC and WSC. The darker the red color is, the higher the dissolution rates are, and the darker the blue color is, the lower the dissolution rates are

**Fig. 7:**
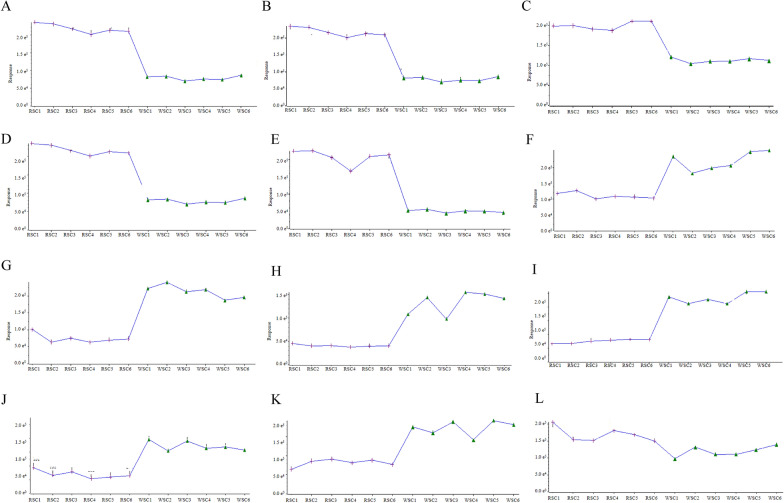
Contents of 12 ingredients in RSC before and after wine steaming. **A** Schizantherin B. **B** Citric acid. **C** Malic acid. **D** Nigranoic acid. **E** Catechin. **F** Schisandrin A. **G** Schisandrin B. **H** Schisanhenol. **I** Gomisin D. **J** Schisandrol B. **K** Schisandrin. **L** Schisantherin A

There were differences between all the components of WSC and RSC (Fig. 6A, B). In the loading plot, the X-axis represents the first principal component, and the Y-axis represents the second principal component (Fig. 6C, D). The higher the absolute values of the principal components are, the more significant the difference between compounds is. The aforementioned method was used to identify 12 compounds with various dissolution rates (Figs. 6E and 7). Specifically, wine-steam processing produced an increase in the dissolution rates of Schisandrin A, Schisandrin B, Schisanhenol, Gomisin D, Schisandrol B, and Schisandrin trend and a decrease in the dissolution rates of Schisantherin B, Citric acid, Malic acid, Nigranoic acid, Catechin, and Schisantherin A.

### Determination of dissolution rates of Schisandrin, Schisandriol B, Schisandrin A, Schisandrin B, Schisanhenol, and Gomisin D

The standard curve for the injection concentration versus the peak area was plotted to obtain a regression equation. The regression equation exhibited excellent linearity within the linear range (Additional file 2: Table S2). The RSDs for the precision and stability were both < 2%, indicating that the six compounds were stable within 24 h and that the determination method was precise. In the repeatability test, the RSDs of all the samples were found to be < 2%. The recoveries of schisandrin, schisandriol B, schisandrin A, schisandrin B, schisanhenol, and gomisin D were 99.56%, 99.34%, 99.27%, 99.83%, 100.20%, and 99.82%, respectively, whereas the RSDs were < 2%, indicating that the established method had good stability and accuracy (Additional file 2: Table S3−S6).

**Fig. 8:**
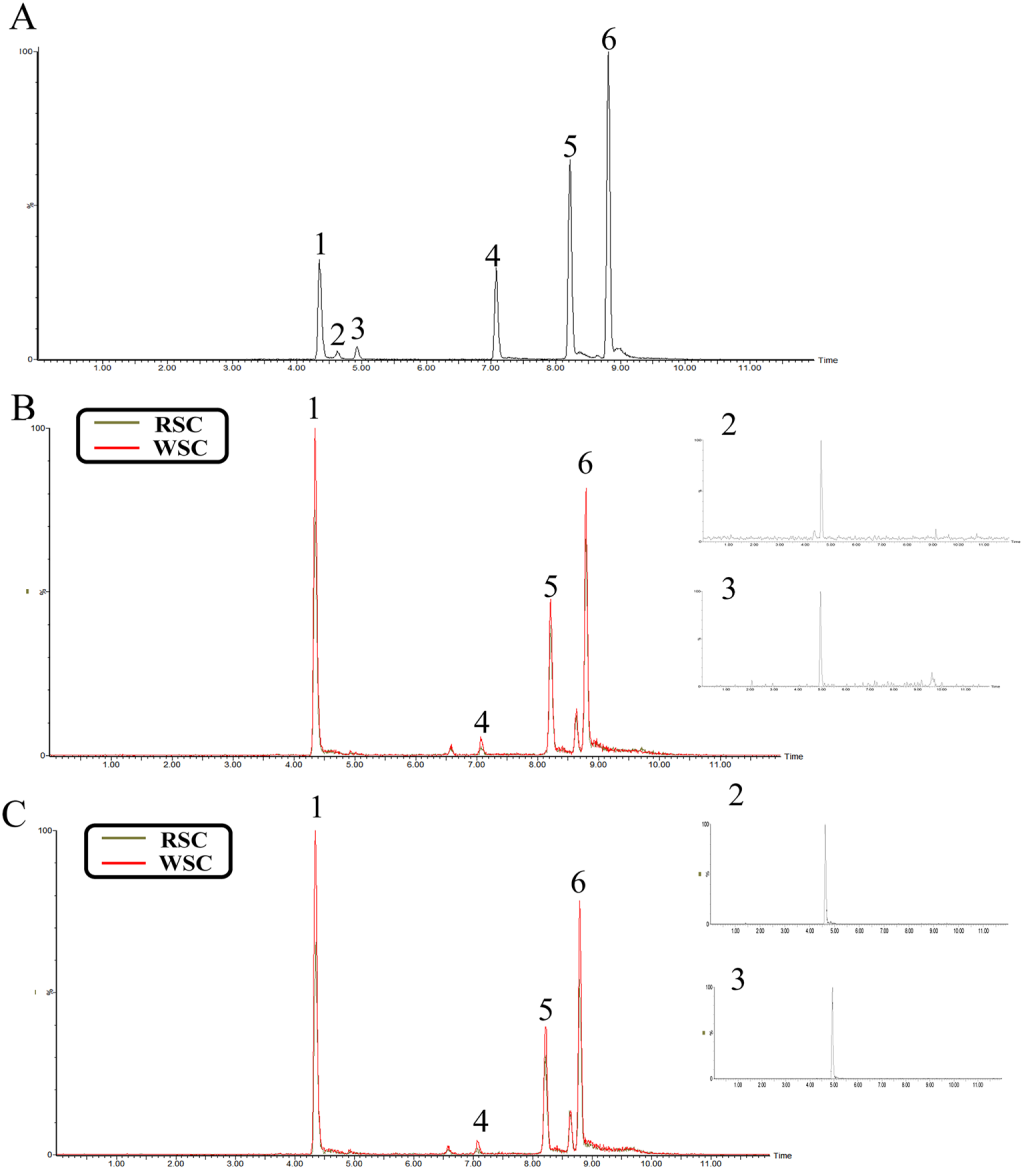
Total ion current diagrams for RSC and WSC obtained using MRM. **A** Reference mixture. **B** Superposition diagram of the total ion currents of RSC and WSC decoctions. **C** Superposition diagram of the total ion currents of methanol extracts of RSC and WSC (1. Schisandrin; 2. Gomisin D; 3. Schisandriol B; 4. Schisanhenol; 5. Schisandrin A; 6. Schisandrin B)

The total ion current diagram of RSC and WSC obtained using MRM is shown in Fig. 8. Table 3 shows that the contents of Schisandrin, Schisandriol B, Schisandrin A, Schisandrin B, Schisanhenol, and Gomisin D in the RSC decoction were 0.284%, 0.053%, 0.078%, 0.109%, 0.015%, and 0.006%, respectively, and those in the WSC decoction were 0.412%, 0.075%, 0.092%, 0.145%, 0.027%, and 0.025%, respectively. The water dissolution rate was calculated to determine dissolution rates of Schisandrin, Schisandriol B, Schisandrin A, Schisandrin B, Schisanhenol, and Gomisin D in the RSC decoction of 35.375%, 36.205%, 32.564%, 33.273%, 27.861%, and 17.626%, respectively, and those in the WSC decoction as 46.939%, 46.652%, 35.837%, 39.940%, 42.095%, and 36.890%, respectively.

**Table 3:** The results of content determination and water dissolution rates of 6 components in RSC and WSC

Term of determination	Samples	Schisandrin (%)	Schisandrol B (%)	Schisandrin A (%)	Schisandrin B (%)	Schisanhenol (%)	Gomisin D (%)
Content in decoction	RSC	0.284	0.053	0.078	0.109	0.015	0.006
	WSC	0.412	0.075	0.092	0.145	0.027	0.025
Content of methanol extract	RSC	0.802	0.145	0.242	0.330	0.055	0.033
	WSC	0.879	0.161	0.258	0.363	0.064	0.068
Dissolution rate	RSC	35.375	36.205	32.564	33.273	27.861	17.626
	WSC	46.939	46.652	35.837	39.940	42.095	36.890

The quantitative verification results showed that the dissolution rate of these six components was indeed increased by wine steaming. Considering the potential pharmacodynamically active components of WSC, we speculated that the improvement in the efficacy of WSC might be related to the increase in the dissolution rates of Schisandrin, Schisandriol B, Schisandrin A, Schisandrin B, and Gomisin D. Therefore, we preliminarily selected Schisandrin, Schisandriol B, Schisandrin A, Schisandrin B, and Gomisin D as Q-markers for the efficacy of WSC.

### In vivo efficacy validation

#### MTC measurement results

**Fig. 9:**
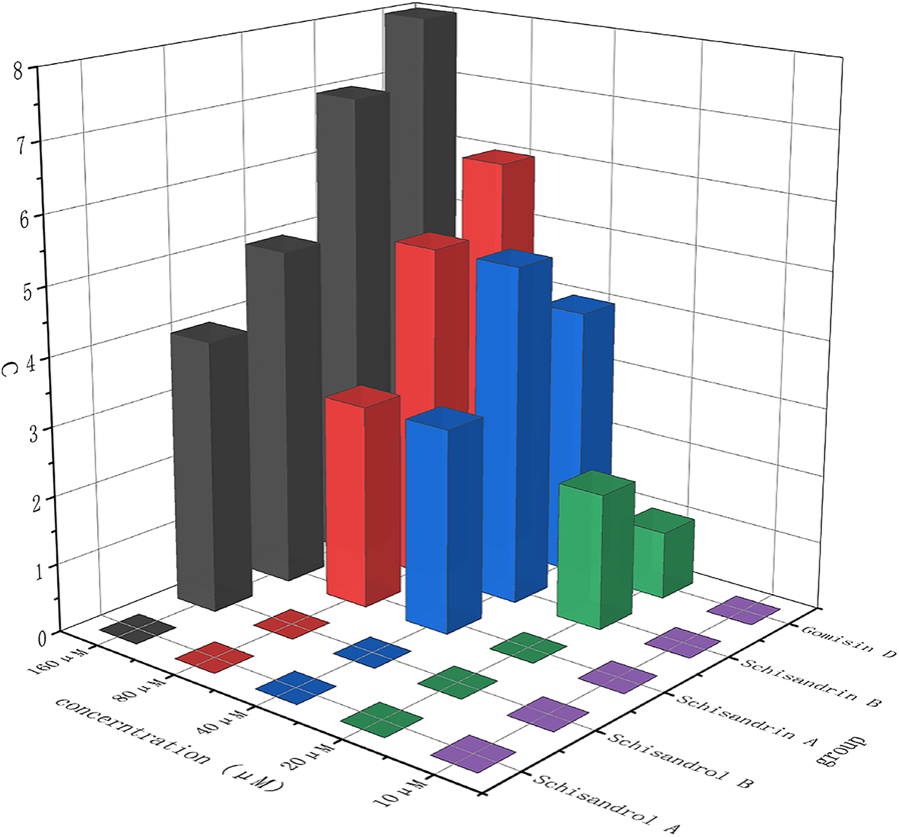
MTC of Schisandrin, Schisandrol B, Schisandrin A, Schisandrin B, and Gomisin D in zebrafish

According to the MTC results for RSC and WSC, the mortality caused by RSC at concentrations of 390 μg/mL, 780 μg/mL, 1560 μg/mL, 3120 μg/mL, and 6240 μg/mL was 0%, 0%, 30%, 50%, and 70%, respectively. The mortality caused by WSC at different concentrations was 0%, 0%, 20%, 40%, and 50%. Therefore, 1560 μg/mL was taken as the MTC of RSC and WSC. Figure 9 shows the dosage determined according to the MTC of each component. Finally, the MTCs of Schisandrin, Schisandriol B, Schisandrin A, Schisandrin B, and Gomisin D were determined to be 160 μM, 80 μM, 20 μM, 10 μM, and 10 μM, respectively.

#### Screening of administration time points

**Fig. 10:**
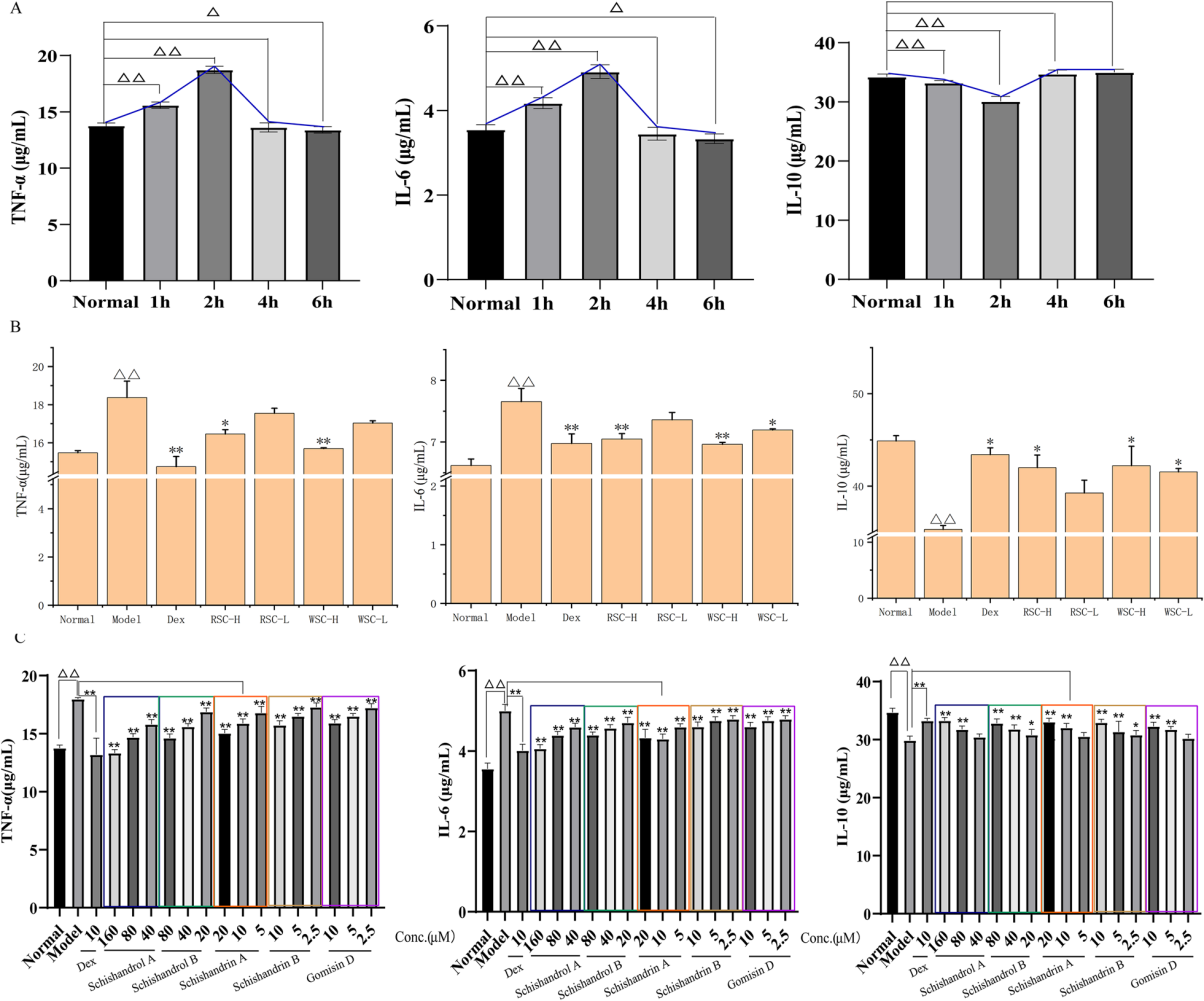
Efficacy validation based on the zebrafish inflammatory model. **A** Screening of the optimal time points. **B** Effects of RSC and WSC on the expression of TNF-α, IL-6, and IL-10. **C** Effects of five Q-markers on the expression of TNF-α, IL-6, and IL-10. The data are expressed as the mean ± SD (n = 10). ^△^*p* < 0.05 and ^△△^*p* < 0.01 vs. normal group, *p < 0.05 and **p < 0.01 vs. model group

ELISA showed that compared with the control group, TNF-α and IL-6 expression in the 1-h and 2-h groups were elevated and the expression of IL-10 was decreased (Fig. 10A). The expression of the indicators was more prominent in the 2-h group (*P* < 0.01). However, there were no noticeable differences in the levels of TNF-α, IL-6, and IL-10 among the 4-h, 6-h, and normal groups. Therefore, 2 h after tail amputation was finally selected as the deadline for treatment administration.

#### Efficacy comparison results

Figure 10B, C shows that compared with the normal group, the expression of TNF-α and IL-6 in the model group was prominently elevated (*P* < 0.01) and the content of IL-10 was conspicuously reduced (*P* < 0.01), demonstrating that the zebrafish inflammation model was successfully established and the high credibility of the research results obtained using this model. However, treatment with RSC, WSC, and all monomer components dramatically reduced the increase in TNF-α and IL-6 expression caused by tail amputation and simultaneously increased the level of IL-10 (*P* < 0.01; *P* < 0.05). The effect of treatment in the WSC-H on reducing the expression of TNF-α and IL-6 was found to be superior to that in RSC-H (no significant difference). In addition, only WSC in the low-dose group could apparently increase the expression of IL-10, suggesting a stronger anti-inflammatory effect of WSC than RSC at the same dose (Fig. 10B). Figure 10C shows that all five monomer components exerted anti-inflammatory effects in a dose-dependent manner. The higher the administered dose was, the stronger the effect of the component on the expression of TNF-α, IL-6, and IL-10 was. Considering these results in combination with previous reports that RSC can treat asthma by anti-inflammatory effects [16] suggested that Schisandrin, Schisandriol B, Schisandrin A, Schisandrin B, and Gomisin D had an anti-inflammatory effect, implying that the improvement in the efficacy of WSC was related to the 5 Q-markers.

## Discussion

RSC is the main herb that ancient and modern doctors have prescribed to treat asthma [28]. In the Synopsis of the Golden Chamber published in the Han Dynasty, RSC was commonly used in prescriptions, such as the Xiaoqinglong [29] and Sheganmahuang [30] decoctions, for warming the lung to dissipate phlegm with the function of restraining lung Qi and relieving cough [31]. RSC is often steamed with wine to enhance its efficiency in clinical applications. Yellow rice wine is one of the commonly used adjuvants in processing and can promote drug blood circulation and accelerate the release of effective components [32]. However, the Q-markers for the efficacy of WSC decoction pieces are still unclear, and a specific quality standard for WSC has not been established.

OVA can cause significant inflammation of the allergic airway and is therefore frequently used in asthma modeling [33]. In this study, an allergic asthma model was established mainly by stimulation with OVA, supplemented with aluminum hydroxide. The rats in the model group exhibited similar asthma symptoms, such as shortness of breath, wheezing, and sneezing. Considering that asthma is marked by lung inflammation, the pathological status of the lungs was evaluated [34]. In the model group, we observed marked inflammatory infiltration in the lungs with thickening of the bronchial wall (Fig. 2C). These results were in accordance with those reported in previous studies[35, 36], suggesting that pathological changes occurred in the tissues in the model group corresponding to asthma. To illustrate the establishment of the model, we investigated the expression of IgE, IFN-γ and IL-4 in the model group. The level of IFN-γ decreased and the levels of IL-4 and IgE increased in the model group. The overexpression of Th2 cytokines, such as IL-4, and the reduced expression of Th1 cytokines, such as IFN-γ, are the main manifestations of a Th1/Th2 imbalance, which is the key mechanism of an asthma attack [37, 38]. Therefore, it is speculated that the model group had a Th1/Th2 imbalance. Increased levels of IgE and other inflammatory factors is also an important cause of the development of asthma [39]. The inflammatory infiltration phenomenon observed in the lungs confirmed the successful establishment of an allergic asthma model, which could therefore be used to compare the efficacy of RSC and WSC. Efficacy studies showed that both RSC and WSC induced an increase in IFN-γ levels and a decrease in IL-4 and IgE levels and improved lung inflammation (Fig. 2B, C), suggesting that these substances could play a role in the treatment of asthma by regulating the imbalance of the Th1/Th2 ratio and reducing IgE levels. In addition, WSC was more potent than RSC at the same dose in regulating the Th1/Th2 imbalance (Fig. 2B). However, the effects of RSC and WSC on reducing IgE secretion were basically the same. Considering that the allergic asthma model established in this study is a classic highly credible model and that the dosages of RSC and WSC comply with clinical requirements, the outcomes obtained in this study are expected to have reference value for clinical practice and provide data support for the application of WSC to the clinical treatment of asthma.

The results presented above show that WSC has a stronger anti-asthma effect than RSC, which has research and application value. Serum pharmacochemistry, a method based on the theory that "the components absorbed and metabolized in blood are the potential active components", was used to determine the bioactive components of WSC [40–42]. Our results demonstrated that all the bioactive components of WSC were prototype components or metabolic components of lignans (consisting of 10 prototypes and 12 metabolites) (Table 1 and Fig. 3).

A quality evaluation standard of RSC with schisandrin A as the index is included in the Chinese Pharmacopoeia, but few studies have been performed on WSC and a specific quality standard evaluation method has not been established. The results of this study demonstrated the excellent efficacy of WSC. Towards developing methods for quality inspection of WSC based on an evaluation of the components, UPLC-Q-TOF–MS/MS was used to identify the common and different chemical components between the WSC and RSC decoction pieces to screen exclusive Q-markers of WSC. A total of 32 common components between the RSC and WSC decoction pieces were found, of which processing changed the dissolution rates of 12 components (Table 2, Figs. 6 , 7 and 8). Combined with the analysis results of components absorbed into the blood of WSC-H group, it was found that 5 components in WSC, Schisandrin, Schisandriol B, Schisandrin A, Schisandrin B, and Gomisin D, had higher dissolution rates than RSC, which may explain the superior efficacy of WSC over RSC. Therefore, we preliminarily designated these 5 components as Q-markers of WSC efficacy.

Asthma is a chronic inflammatory disorder of the airways in which many inflammatory mediators, cytokines, and adhesion molecules play a role [43]. Increased secretion of Th2-type cytokines, such as IL-4, IL-5, and IL-13, in the allergic airway results in increased recruitment of inflammatory cells and airway hyperresponsiveness [44], suggesting that airway inflammation may be related to the severity of asthma. Therefore, we examined the efficacy of the 5 components in terms of their anti-inflammatory abilities [45]. A zebrafish inflammation model was successfully established, as demonstrated by elevated TNF-α and IL-6 expression and reduced expression of IL-10 (Fig. 10A) [46]. In addition, WSC was superior to RSC in reducing TNF-α and IL-6 expression and increasing IL-10 expression at the same dose (there was no significant difference between the results for WSC and RSC), suggesting that WSC had a stronger anti-inflammatory effect than RSC (Fig. 10B). The results of monomer verification experiments showed that all 5 components exerted anti-inflammatory effects in a dose-dependent manner, which together with previous reports that RSC and WSC can treat asthma by anti-inflammatory effects (Fig. 10C) [16] implied that Schisandrin, Schisandriol B, Schisandrin A, Schisandrin B, and Gomisin D were Q-markers of the efficacy of the decoction pieces.

## Conclusion

In this study, Q-markers related to WSC efficacy were screened by pharmacodynamic comparison-component screening-component validation in vivo efficacy. The decoction pieces of WSC had superior efficacy to RSC in improving allergic asthma at the same dose based on the pharmacodynamic comparative analysis. Twelve components of WSC were detected in blood, among which 5 components, Schisandrin, Schisandriol B, Schisandrin A, Schisandrin B, and Gomisin D, have increased dissolution in water decoction after steaming with wine. The in vivo efficacy of these 5 components was verified, that is, these components were Q-markers of the superior efficacy of WSC decoction pieces. This study provides theoretical support for the establishment of specific quality evaluation standards for WSC decoction pieces.

## Supplementary Information

**Additional file 1: Fig. S1** Total ion chromatography of normal and WSC serum samples obtained in positive mode after treatment with methanol and acetonitrile, respectively. **A** Total ion chromatography of the serum samples in the normal group obtained in positive ion mode after treatment with methanol. **B** Total ion chromatography of the serum samples in the normal group obtained in positive ion mode after treatment with acetonitrile. **C** Total ion chromatography of the serum samples in the WSC-H group obtained in positive ion mode after treatment with methanol. **D** Total ion chromatography of the serum samples in the WSC-H group obtained in positive ion mode after treatment with acetonitrile. Refer to the text for detailed analysis conditions, and the compounds labeled in the figure correspond to Table 1. **Fig. S2. **Total ion chromatography of normal and WSC serum samples obtained in negative mode after treatment with methanol and acetonitrile, respectively. **A** Total ion chromatography of the serum samples in the normal group obtained in negative ion mode after treatment with methanol. **B** Total ion chromatography of the serum samples in the normal group obtained in negative ion mode after treatment with acetonitrile. **C** Total ion chromatography of the serum samples in the WSC-H group obtained in negative ion mode after treatment with methanol. **D** Total ion chromatography of the serum samples in the WSC-H group obtained in negative ion mode after treatment with acetonitrile.

**Additional file 2:** **Table S1**. Mass spectrometric parameters of fifteen components. **Table S2**. Investigation of linear relation. **Table S3**. Precision test results. **Table S4**. Stability test results. **Table S5. **Repeatability test results. **Table S6.** Experimental results of sample recovery.

## Data Availability

The datasets used and/or analyzed during the current study are available from the corresponding author on reasonable request.

## Abbreviations

TCM: Traditional Chinese medicine
WSC: Wine-steamed Schisandra Chinensis Fructus
RSC: Raw Schisandra Chinensis Fructus
UPLC-Q-TOF-MS/MS: Ultra-performance liquid chromatography quadrupole time-of-flight mass spectrometry
UPLC H-CLASS XEVO TQD: Ultra-high-performance liquid chromatography tandem mass spectrometry
Q-markers: Quality markers
WSC-H: High dose group of WSC
Dex: Dexamethasone
IgE: Immunoglobulin E
IFN-γ: Interferon γ
IL-4: Interleukin 4
HE: Hematoxylin–eosin
PCA: Principal component analysis
MTC: Maximum tolerance concentration
TNF-α: Tumor necrosis factor-α
IL-6: Interleukin 6
IL-10: Interleukin 10
