# Supplementary material for: Screening of Q-markers for the wine-steamed Schisandra chinensis decoction pieces in improving allergic asthma
Source: Chin Med. 2023 Jan 30;18:10. doi: 10.1186/s13020-023-00712-0 (PMC9887854; doi:10.1186/s13020-023-00712-0)
Supplement: Supplementary file 1 — Additional file 1: Fig. S1 Total ion chromatography of normal and WSC serum samples obtained in positive mode after treatment with methanol and acetonitrile, respectively. A Total ion chromatography of the serum samples in the normal group obtained in positive ion mode after treatment with methanol. B Total ion chromatography of the serum samples in the normal group obtained in positive ion mode after treatment with acetonitrile. C Total ion chromatography of the serum samples in the WSC-H group obtained in positive ion mode after treatment with methanol. D Total ion chromatography of the serum samples in the WSC-H group obtained in positive ion mode after treatment with acetonitrile. Refer to the text for detailed analysis conditions, and the compounds labeled in the figure correspond to Table 1. Fig. S2. Total ion chromatography of normal and WSC serum samples obtained in negative mode after treatment with methanol and acetonitrile, respectively. A Total ion chromatography of the serum samples in the normal group obtained in negative ion mode after treatment with methanol. B Total ion chromatography of the serum samples in the normal group obtained in negative ion mode after treatment with acetonitrile. C Total ion chromatography of the serum samples in the WSC-H group obtained in negative ion mode after treatment with methanol. D Total ion chromatography of the serum samples in the WSC-H group obtained in negative ion mode after treatment with acetonitrile. [file 13020_2023_712_MOESM1_ESM.docx]

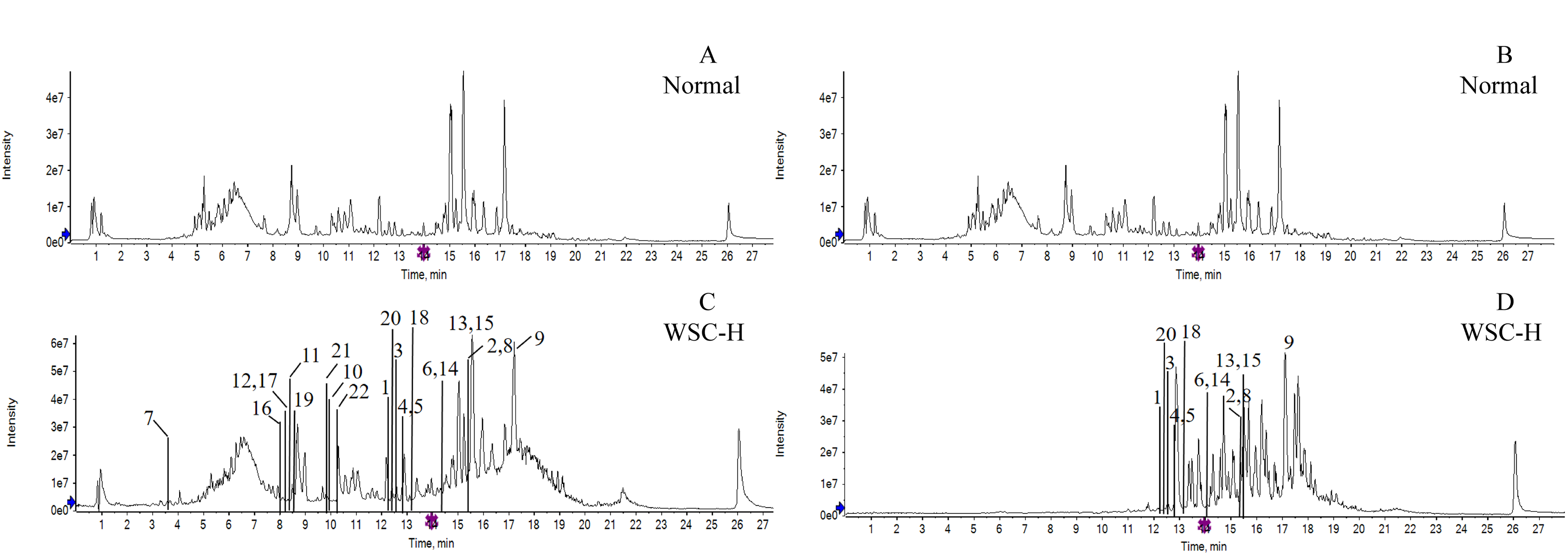


**Fig. S1** Total ion chromatography of normal and WSC serum samples obtained in positive mode after treatment with methanol and acetonitrile, respectively. **A** Total ion chromatography of the serum samples in the normal group obtained in positive ion mode after treatment with methanol. **B** Total ion chromatography of the serum samples in the normal group obtained in positive ion mode after treatment with acetonitrile. **C** Total ion chromatography of the serum samples in the WSC-H group obtained in positive ion mode after treatment with methanol. **D** Total ion chromatography of the serum samples in the WSC-H group obtained in positive ion mode after treatment with acetonitrile. Refer to the text for detailed analysis conditions, and the compounds labeled in the figure correspond to Table 1.

**
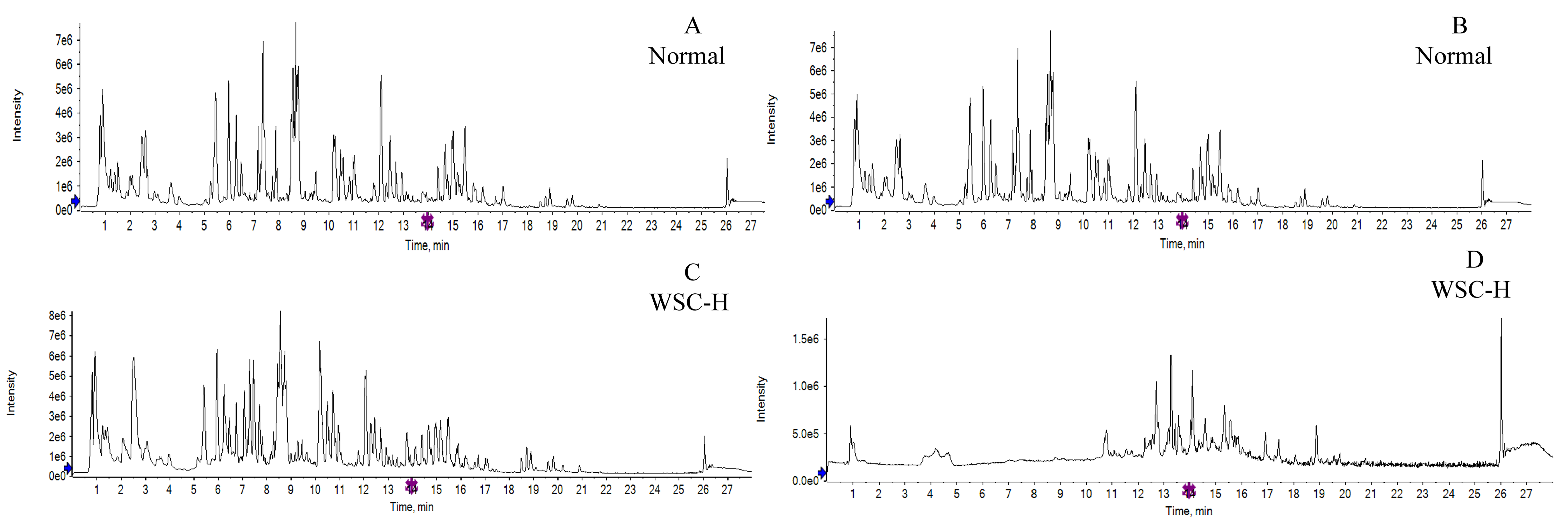
**

**Fig. S2.** Total ion chromatography of normal and WSC serum samples obtained in negative mode after treatment with methanol and acetonitrile, respectively. **A** Total ion chromatography of the serum samples in the normal group obtained in negative ion mode after treatment with methanol. **B** Total ion chromatography of the serum samples in the normal group obtained in negative ion mode after treatment with acetonitrile. **C** Total ion chromatography of the serum samples in the WSC-H group obtained in negative ion mode after treatment with methanol. **D** Total ion chromatography of the serum samples in the WSC-H group obtained in negative ion mode after treatment with acetonitrile.
