# Supplementary material for: Screening of Q-markers for the wine-steamed Schisandra chinensis decoction pieces in improving allergic asthma
Source: Chin Med. 2023 Jan 30;18:10. doi: 10.1186/s13020-023-00712-0 (PMC9887854; doi:10.1186/s13020-023-00712-0)
Supplement: Supplementary file 2 — Additional file 2: Table S1. Mass spectrometric parameters of fifteen components. Table S2. Investigation of linear relation. Table S3. Precision test results. Table S4. Stability test results. Table S5. Repeatability test results. Table S6. Experimental results of sample recovery. [file 13020_2023_712_MOESM2_ESM.docx]

**Table S1**. Mass spectrometric parameters of fifteen components.

| Compound | Formula | RT  (min) | ion pair  (*m/z*) | DP/V | CE/V | Ionic mode |
| --- | --- | --- | --- | --- | --- | --- |
| Schisandrin | C_24_H_32_O_7_ | 4.3985 | 433.32/415.34 | 31 | 10 | ESI^+^ |
| Schisandrol B | C_23_H_28_O_7_ | 4.9256 | 417.12/399.24 | 55 | 7 | ESI^+^ |
| Schisandrin A | C_24_H_32_O_6_ | 8.3027 | 417.31/316.18 | 56 | 17 | ESI^+^ |
| Schisandrin B | C_23_H_28_O_6_ | 8.7564 | 401.32/300.15 | 50 | 25 | ESI^+^ |
| Schisanhenol | C_23_H_30_O_6_ | 6.8537 | 403.24/340.21 | 60 | 20 | ESI^+^ |
| Gomisin D | C_28_H_34_O_10_ | 4.5792 | 553.25/507.32 | 70 | 25 | ESI^+^ |

**Table S2**. Investigation of linear relation.

| Composition | Regression equation | R | Linear range (ng/mL) |
| --- | --- | --- | --- |
| Schisandrin | Y=146388x-12.303 | 0.9997 | 20~640 |
| Schisandrol B | Y=6825X-2.6567 | 0.9996 | 2.5~80 |
| Schisandrin A | Y=269409X+269.38 | 0.9990 | 6.25~200 |
| Schisandrin B | Y=266803X+948.86 | 0.9997 | 10.31~330 |
| Schisanhenol | Y=142638X+45.681 | 0.9991 | 0.72~25 |
| Gomisin D | Y=4313.8X+1.792 | 0.9990 | 0.653~21 |

**Table S3**. Precision test results.

| No. | Schisandrin | | Schisandrol B | | Schisandrin A | | Schisandrin B | | Schisanhenol | | Gomisin D | |
| --- | --- | --- | --- | --- | --- | --- | --- | --- | --- | --- | --- | --- |
|  | RT  (min) | Peak area | RT  (min) | Peak area | RT  (min) | Peak area | RT  (min) | Peak area | RT  (min) | Peak area | RT  (min) | Peak area |
| 1 | 4.4361 | 6356.5268 | 4.9382 | 56.8021 | 8.2514 | 2676.7899 | 8.7254 | 8917.5308 | 6.6537 | 418.6697 | 4.6539 | 12.4661 |
| 2 | 4.3582 | 6148.6193 | 4.8951 | 56.4771 | 8.2159 | 2701.6324 | 8.7943 | 8808.7662 | 6.8529 | 433.4264 | 4.6264 | 12.2157 |
| 3 | 4.3985 | 6024.1982 | 4.9256 | 58.46625 | 8.3027 | 2760.5879 | 8.7564 | 8817.5681 | 6.8537 | 425.1506 | 4.5792 | 12.7491 |
| 4 | 4.3854 | 6250.5376 | 4.9074 | 57.6407 | 8.1547 | 2686.0215 | 8.7325 | 8730.2716 | 6.8539 | 436.8274 | 4.6158 | 12.2695 |
| 5 | 4.3544 | 6040.3372 | 4.9537 | 56.18475 | 8.2016 | 2739.6773 | 8.759 | 8815.2992 | 6.9503 | 427.7456 | 4.6539 | 12.1876 |
| 6 | 4.3985 | 6072.0564 | 4.9628 | 56.23915 | 8.3084 | 2695.8412 | 8.7359 | 8770.8472 | 6.8539 | 442.0268 | 4.6358 | 12.8054 |
| RSD（%） | 0.6279 | 1.9548 | 0.4864 | 1.4529 | 0.6660 | 1.1058 | 0.2636 | 1.1864 | 1.3019 | 1.7924 | 0.5533 | 2.0011 |

**Table S4**. Stability test results.

| No. | Schisandrin | | Schisandrol B | | Schisandrin A | | Schisandrin B | | Schisanhenol | | Gomisin D | |
| --- | --- | --- | --- | --- | --- | --- | --- | --- | --- | --- | --- | --- |
|  | RT  (min) | Peak area | RT  (min) | Peak area | RT  (min) | Peak area | RT  (min) | Peak area | RT  (min) | Peak area | RT  (min) | Peak area |
| 0 h | 4.5536 | 6094.5178 | 4.9354 | 53.16085 | 8.3567 | 2833.1508 | 8.7036 | 4500.5648 | 6.8159 | 447.6826 | 4.6539 | 12.66365 |
| 2 h | 4.3762 | 6139.2619 | 4.912 | 54.8266 | 8.2317 | 2737.3291 | 8.7126 | 4530.1763 | 6.9032 | 432.5157 | 4.6264 | 12.77805 |
| 4 h | 4.3582 | 5987.8115 | 4.937 | 53.26595 | 8.2164 | 2798.3187 | 8.7452 | 4436.8266 | 6.8035 | 442.5263 | 4.6532 | 12.43965 |
| 6 h | 4.398 | 6054.6632 | 4.9532 | 53.82125 | 8.1736 | 2782.5291 | 8.7024 | 4556.0029 | 6.8143 | 432.8557 | 4.6218 | 12.68435 |
| 12 h | 4.3467 | 6151.3178 | 4.9671 | 53.94775 | 8.2654 | 2678.8217 | 8.7136 | 4559.1608 | 6.8536 | 446.3157 | 4.7356 | 12.20795 |
| 24 h | 4.3265 | 6151.7937 | 4.9135 | 52.8774 | 8.2142 | 2712.4485 | 8.7603 | 4567.2598 | 6.8217 | 433.9432 | 4.6274 | 12.89625 |
| RSD（%） | 1.7103 | 0.9809 | 0.4005 | 1.1998 | 0.6988 | 1.9101 | 0.2512 | 1.0011 | 0.4982 | 1.4579 | 0.9340 | 1.8000 |

**Table S5.** Repeatability test results.

| No. | Schisandrin | | Schisandrol B | | Schisandrin A | | Schisandrin B | | Schisanhenol | | Gomisin D | |
| --- | --- | --- | --- | --- | --- | --- | --- | --- | --- | --- | --- | --- |
|  | RT  (min) | Content(%) | RT  (min) | Content(%) | RT  (min) | Content(%) | RT  (min) | Content(%) | RT  (min) | Content(%) | RT  (min) | Content(%) |
| 1 | 4.3751 | 0.4137 | 4.9639 | 0.0743 | 8.2539 | 0.1003 | 8.7064 | 0.1430 | 6.8539 | 0.0308 | 4.6237 | 0.0257 |
| 2 | 4.3762 | 0.4176 | 4.9537 | 0.0738 | 8.1357 | 0.0986 | 8.7359 | 0.1398 | 6.8321 | 0.0308 | 4.6848 | 0.0250 |
| 3 | 4.3129 | 0.4185 | 4.9321 | 0.0742 | 8.3257 | 0.0985 | 8.7154 | 0.1468 | 6.8126 | 0.0310 | 4.6579 | 0.0257 |
| 4 | 4.3796 | 0.4320 | 4.9035 | 0.0752 | 8.2754 | 0.0978 | 8.7439 | 0.1424 | 6.8537 | 0.0299 | 4.6314 | 0.0264 |
| 5 | 4.3056 | 0.4247 | 4.9126 | 0.0746 | 8.3125 | 0.0955 | 8.7531 | 0.1446 | 6.9037 | 0.0313 | 4.6578 | 0.0254 |
| 6 | 4.3179 | 0.4214 | 4.9523 | 0.0756 | 8.3014 | 0.0984 | 8.6945 | 0.1473 | 6.8531 | 0.0306 | 4.6539 | 0.0257 |
| RSD（%） | 0.7513 | 1.3857 | 0.4511 | 0.8489 | 0.7678 | 1.4666 | 0.2401 | 1.7894 | 0.4053 | 1.4198 | 0.4274 | 1.6745 |

**Table S6.** Experimental results of sample recovery.

| Component | Sample content  (mg) | Sample addition  (mg) | Measured value  (mg) | Recovery  (%) | Average recovery  (%) | RSD  (%) |
| --- | --- | --- | --- | --- | --- | --- |
| Schisandrin | 2.8356 | 2.700 | 5.5056 | 98.8 | 99.56 | 1.96 |
|  | 2.6086 | 2.700 | 5.3186 | 100.3 |  |  |
|  | 2.661 | 2.700 | 5.2631 | 96.3 |  |  |
|  | 2.7453 | 2.700 | 5.5235 | 102.8 |  |  |
|  | 2.6419 | 2.70 | 5.3119 | 98.9 |  |  |
|  | 2.7058 | 2.700 | 5.4058 | 100.0 |  |  |
| Schisandrol B | 0.5255 | 0.520 | 1.0415 | 99.2 | 99.34 | 1.45 |
|  | 0.5382 | 0.520 | 1.0458 | 97.6 |  |  |
|  | 0.5069 | 0.520 | 1.0296 | 100.5 |  |  |
|  | 0.5137 | 0.520 | 1.0273 | 98.7 |  |  |
|  | 0.5248 | 0.520 | 1.0544 | 101.8 |  |  |
|  | 0.5465 | 0.520 | 1.0567 | 98.1 |  |  |
| Schisandrin A | 0.7866 | 0.770 | 1.5554 | 99.8 | 99.27 | 1.58 |
|  | 0.7931 | 0.770 | 1.5406 | 97.1 |  |  |
|  | 0.7542 | 0.770 | 1.5318 | 100.9 |  |  |
|  | 0.785 | 0.770 | 1.5455 | 98.7 |  |  |
|  | 0.7792 | 0.770 | 1.5314 | 97.6 |  |  |
|  | 0.7463 | 0.770 | 1.5261 | 101.2 |  |  |
| Schisandrin B | 1.0992 | 1.100 | 2.1792 | 98.2 | 99.83 | 1.91 |
|  | 1.1253 | 1.100 | 2.2325 | 100.6 |  |  |
|  | 1.0694 | 1.100 | 2.1496 | 98.2 |  |  |
|  | 1.2267 | 1.100 | 2.3672 | 103.6 |  |  |
|  | 1.0549 | 1.100 | 2.1459 | 99.2 |  |  |
|  | 1.1784 | 1.100 | 2.2687 | 99.2 |  |  |
| Schisanhenol | 0.1524 | 0.150 | 0.3035 | 100.7 | 100.2 | 1.60 |
|  | 0.1569 | 0.150 | 0.3053 | 98.9 |  |  |
|  | 0.1487 | 0.150 | 0.3019 | 102.1 |  |  |
|  | 0.1569 | 0.150 | 0.3032 | 97.5 |  |  |
|  | 0.1492 | 0.150 | 0.3019 | 101.8 |  |  |
|  | 0.1538 | 0.150 | 0.3038 | 100.0 |  |  |
| Gomisin D | 0.0581 | 0.057 | 0.1154 | 100.5 | 99.82 | 1.76 |
|  | 0.0579 | 0.057 | 0.1142 | 98.7 |  |  |
|  | 0.0584 | 0.057 | 0.1138 | 97.2 |  |  |
|  | 0.0586 | 0.057 | 0.1155 | 99.8 |  |  |
|  | 0.0592 | 0.057 | 0.1160 | 99.6 |  |  |
|  | 0.0588 | 0.057 | 0.1175 | 102.9 |  |  |
